# Metagenomic and Metabolic Profiling of Nonlithifying and Lithifying Stromatolitic Mats of Highborne Cay, The Bahamas

**DOI:** 10.1371/journal.pone.0038229

**Published:** 2012-05-25

**Authors:** Christina L. M. Khodadad, Jamie S. Foster

**Affiliations:** Department of Microbiology and Cell Science, Space Life Science Lab, University of Florida, Kennedy Space Center, Florida, United States of America; Université Paris Sud, France

## Abstract

**Background:**

Stromatolites are laminated carbonate build-ups formed by the metabolic activity of microbial mats and represent one of the oldest known ecosystems on Earth. In this study, we examined a living stromatolite located within the Exuma Sound, The Bahamas and profiled the metagenome and metabolic potential underlying these complex microbial communities.

**Methodology/Principal Findings:**

The metagenomes of the two dominant stromatolitic mat types, a nonlithifying (Type 1) and lithifying (Type 3) microbial mat, were partially sequenced and compared. This deep-sequencing approach was complemented by profiling the substrate utilization patterns of the mats using metabolic microarrays. Taxonomic assessment of the protein-encoding genes confirmed previous SSU rRNA analyses that bacteria dominate the metagenome of both mat types. Eukaryotes comprised less than 13% of the metagenomes and were rich in sequences associated with nematodes and heterotrophic protists. Comparative genomic analyses of the functional genes revealed extensive similarities in most of the subsystems between the nonlithifying and lithifying mat types. The one exception was an increase in the relative abundance of certain genes associated with carbohydrate metabolism in the lithifying Type 3 mats. Specifically, genes associated with the degradation of carbohydrates commonly found in exopolymeric substances, such as hexoses, deoxy- and acidic sugars were found. The genetic differences in carbohydrate metabolisms between the two mat types were confirmed using metabolic microarrays. Lithifying mats had a significant increase in diversity and utilization of carbon, nitrogen, phosphorus and sulfur substrates.

**Conclusion/Significance:**

The two stromatolitic mat types retained similar microbial communities, functional diversity and many genetic components within their metagenomes. However, there were major differences detected in the activity and genetic pathways of organic carbon utilization. These differences provide a strong link between the metagenome and the physiology of the mats, as well as new insights into the biological processes associated with carbonate precipitation in modern marine stromatolites.

## Introduction

Stromatolites are laminated deposits of calcium carbonate formed by the metabolic activities of microbial mats. Stromatolites have a long fossil record, dating back to over 3.5 billion years and represent one of Earth’s earliest known ecosystems [1], [2]. Currently, there are only a few known locations where active, stromatolite-forming microbial mats occur, one of which is the island of Highborne Cay located within the Exuma Sound, The Bahamas.

**Figure 1:**
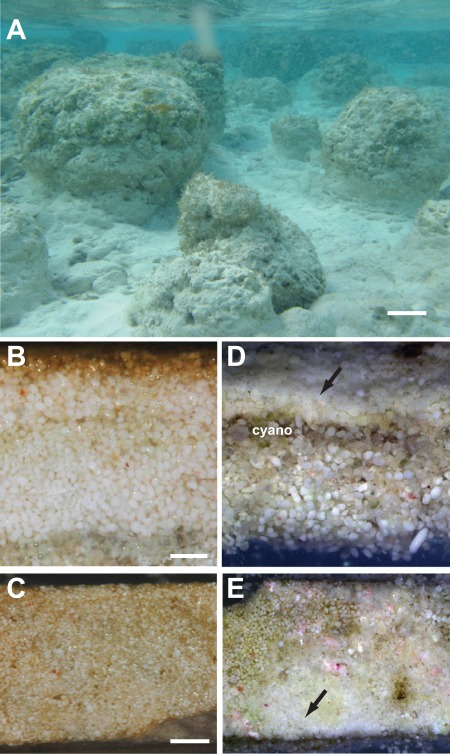
Stromatolites of Highborne Cay, The Bahamas. A. Underwater images of stromatolite build-ups in the subtidal zone. Bar = 10 cm. B. Cross section of a nonlithifying Type 1 stromatolitic mat showing extensive exopolymeric substances (EPS; caramel color) in the upper layer of the mat. Bar = 1 mm. C. Surface of nonlithifying Type 1 mats showed no signs of micritic carbonate deposition in the EPS material (caramel color). Bar = 2 mm. D. Cross section of lithifying Type 3 stromatolitic mat with pronounced layer of sand grains colonized by euendolithic cyanobacteria (cyano), as well as extensive carbonate deposition on the surface (arrow). Bar = 1 mm. E. Surface of lithifying microbial mat with extensive patches of micritic carbonate deposition (arrow). Bar = 2 mm.

For more than a decade the stromatolites of Highborne Cay (Figure 1A) have served as a model to understand the mechanisms of stromatolite formation and development [3]–[7]. In these previous studies three dominant mat communities were identified as contributing to the deposition of the stromatolite microstructure [4], [7]. These mat types are referred to as Type 1, 2, and 3 stromatolitic mats and differ in the bacterial composition and the extent of carbonate mineralization [4], [8]. Type 1 mats are nonlithifying stromatolitic mats enriched in filamentous cyanobacteria, which trap carbonate sand grains. The grains are then actively bound through the secretion of exopolymeric substances (EPS), as the filamentous cyanobacteria move to the sediment surface. The EPS material provides structural scaffolding under high wave activity and a means for microbial adherence (Figure 1B, C) [9]. Type 1 mats are the dominant stromatolitic mat type at Highborne Cay comprising ∼75% of the surface mat communities [10]. Type 2 mats represent a transitional state of stromatolitic mat development and are characterized by a continuous surface film of EPS material interspersed with a thin (20–60 µm) layer of microcrystalline calcium carbonate (i.e., micrite). Type 2 mats are the least abundant stromatolitic mat type (∼5%) at Highborne Cay and are seasonal, forming only in the summer months when both temperature and photosynthetic active radiation (PAR) levels are high [10]. Type 3 mats are lithifying stromatolitic mats characterized by an extensive colonization of the sand grains by euendolithic cyanobacteria (Figure 1D) and a micritic crust on the surface of the mat (Figure 1E) [4], [6]. Under the surface crust, the euendolithic cyanobacteria bore into the sand grains and microbe-induced carbonate precipitation fuses the sand grains together resulting in the formation of a lithified layer of calcium carbonate [6]. Type 3 mats represent 20% of the surface mat communities at Highborne Cay and are most abundant in the summer and fall at Highborne Cay [10].

The cycling between these mat types on the stromatolite surface and the periodic formation of calcium carbonate layers results in the laminated macrostructure of the stromatolites [4]. In other words, each laminae is representative of a former surface mat community and provides a chronology of stromatolite development. Previous analysis of the environmental controls that influence the microbial mat cycling has shown that temperature, PAR, sand burial and abrasion events play an important role in the transitions between surface mat types [4], [10].

The microbial diversity associated with these mat types has been previously characterized using both classic morphology [7] and culture-independent SSU rRNA cloning and sequencing [8], [11], [12]. Results of these previous studies have indicated that the communities are dominated by Proteobacteria, primarily Alphaproteobacteria and Deltaproteobacteria, Cyanobacteria and Bacteroidetes [8], [12]. Although there were few differences between the mat types at the phyla and class-level, previous results indicated that the species richness increased in each mat, with the nonlithifying Type 1 mat having the lowest level of microbial diversity and the lithifying Type 3 having the highest [8]. Biogeochemical analysis also revealed key metabolic differences between the three mat types. The interstitial pH was higher in the lithifying Type 2 (pH 9.4) and 3 (pH 9.2) mats. These mat types exhibited higher rates of photosynthesis and sulfate reduction compared to the nonlithifying Type 1 mat (pH 8.9) [8]. Both of these metabolisms, as well as sulfide oxidation, respiration and fermentation are hypothesized to play key roles in the regulation of net carbonate precipitation and dissolution within the stromatolites [3], [13]–[15].

Although there have been numerous studies on the Bahamian stromatolites, most of this previous work has focused on the bacterial and viral diversity, biogeochemistry and mineralogy [3], [4], [7], [8], [11], [14], [16]. The molecular pathways and functional genes underlying the ecophysiology of this ecosystem remain undescribed. In this study the metabolic potential of the two end members of the Highborne Cay stromatolitic mats, the nonlithifying Type 1 mats and lithifying Type 3 mats were compared using metagenomic sequencing and metabolic phenotypic microarrays. Together, these approaches provide insight into the molecular complexity of the Bahamian stromatolites, as well as correlate the presence of specific taxa to metabolic pathways. Type 2 mats were not included in this study due to their low abundance in the field and biogeochemical and taxonomic similarity to Type 3 mats [8]. Metagenomic sequencing has emerged as a robust means to study the community composition and genomes of complex microbial communities in their natural environments and requires no *a priori* knowledge about the *in situ* genetic material for detection [17]–[19]. Here, we profile the functional and metabolic complexity of the Bahamian stromatolitic mats providing new insight into our understanding of the microbial processes associated with stromatolite formation.

## Results

To examine the functional complexity of the stromatolitic mats a two-pronged approach was used, including metagenomic sequencing of mat genomic DNA, and community physiology testing using metabolic microarrays. Briefly, the total number of high quality sequencing reads recovered from the stromatolitic mats was 71,165 for the nonlithifying mat (Type 1) and 62,744 for the lithifying mat (Type 3) with a mean GC content 41 and 39%, respectively. To normalize the metagenomic data an equalized number of quality pyrosequencing reads (n = 47,520) was randomly selected in triplicate from each mat type and used for several of the downstream analyses (for details see Materials and Methods).

### Community analysis of nonlithifying and lithifying stromatolitic mats

Pyrosequencing results of both the Type 1 and 3 mat types revealed that the majority of the recovered protein-coding sequences were assigned to the domain Bacteria (55.3% Type 1; 51% Type 3). Eukaryota accounted for 7% of the recovered Type 1 reads and 13% of the Type 3 mats. Less than 1% of reads for both mat types were assigned to Archaea. A few viral protein-coding genes were also recovered from the stromatolitic mats representing <0.5% of the stromatolite metagenomes. More than one third of the recovered reads (37%, Type 1; 33% Type 3), however, had no hits within the NCBI-nr database and were unable to be assigned a taxonomic designation within MEGAN.

**Figure 2:**
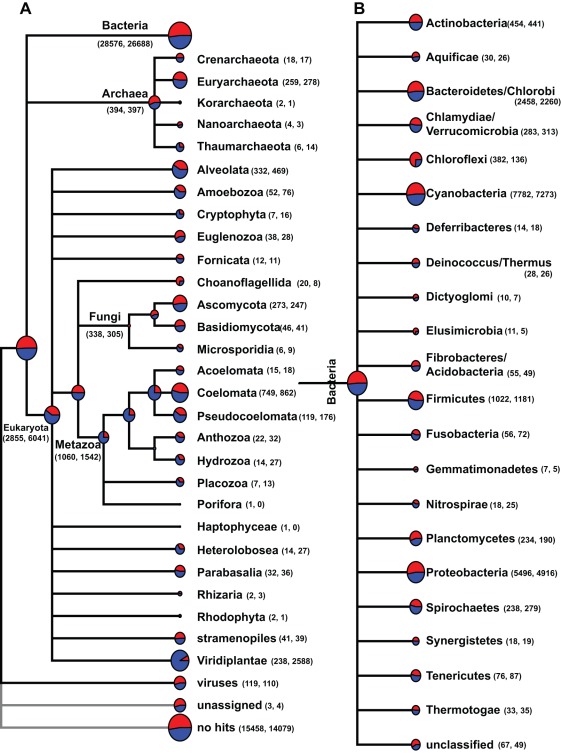
Taxonomic composition of the stromatolite metagenomes using MEGAN analysis. A. Overview of the pyrosequencing reads assigned to the Bacteria, Archaea and Eukaryota. B. Higher resolution of reads associated with the domain Bacteria. Reads derived from nonlithifying Type 1 mats are in red, whereas reads from lithifying Type 3 mats are in blue. The number of reads associated with each taxa are listed in parentheses, with Type 1 and 3 mats listed, respectively. Higher taxa level include unclassified sequences. For example, in the Metazoa many Type 3 (blue) sequences are unable to be assigned beyond the kingdom level.

A comparison of the assigned phylogeny between mat types revealed a broad range of taxa represented in the stromatolitic mat metagenomes (Figure 2). Most of the assigned archaeal sequences were similar to protein-coding genes of Euryarchaeota (66% Type 1; 70% Type 3) with only a few sequences sharing similarity to the Crenarchaeota (4.5% Type 1; 4.3% Type 3) and Thaumarchaeota (1.5% Type 1; 3.5% Type 3). Overall, there were few taxonomic differences in archaeal populations between the nonlithifying and lithifying stromatolitic mats (Figure 2A).

The protein-coding genes assigned to Eukaryota taxa were diverse representing more than a dozen kingdoms, superphyla and phyla (note classification system based on NCBI database) (Figure 2A). Of the various taxa represented in the metagenomes, the Metazoa accounted for 37% of the total Eukaryota reads in Type 1 and 25% in Type 3 mats. Most of the metazoan reads shared similarity to the Coelomata, specifically taxa associated with the Platyhelminthes, Echinodermata, Arthropoda and Pseudocoelomata. In addition to the Metazoa, numerous sequences matched members of various protist superphyla and phyla, most notably the Alveolata (11.6% Type 1; 7.7% Type 3). Other protists represented in the metagenomes include the Amoebozoa, Cryptophyta, Euglenozoa, Heterolobosea, and Parabasalia, comprising a combined 7% in Type 1 and 5% in Type 3 mats. Within the Eukaryota the largest difference between mat types was observed in Viridiplantae, specifically green algae associated with Chlorophyta and Streptophyta. In Type 1 mats Viridiplantae-like sequences accounted for 8.3% of the Eukaryota sequences, whereas in Type 3 mats there was a five-fold increase (43%) in sequences assigned to Streptophyta.

**Figure 3:**
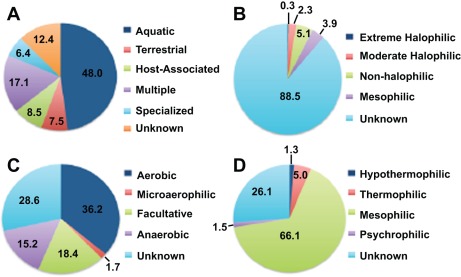
Environmental characteristics of metagenomic sequences. Percentage of sequencing reads associated with A. Habitat. B. Salinity. C. Oxygen tolerance. D. Temperature.

Of the assigned reads, most shared similarity to sequences from Bacteria, representing 25 phyla (Figure 2B). The majority of the observed phyla have been previously reported in Bahamian stromatolites through analysis of 16S rRNA clone libraries [8], [12]. As in these previous microbial diversity analyses, the dominant bacterial phyla in both mat metagenomes were Cyanobacteria (27% Type 1 and 3) and Proteobacteria (19% Type 1; 18% Type 3). Within the Cyanobacteria, most of the recovered sequences were assigned to the order Chroococcales (59% Type 3; 61% Type 3) and Oscillatoriales (25% Type 1; 23% Type 3) (supplemental Figure S1). Surprisingly, 15% of the recovered cyanobacterial sequences from Type 1 and 14% from Type 3 mats shared similarity to Nostocales, which had not been previously detected in Bahamian stromatolites using 16S rRNA sequencing [12]. Most of the recovered Proteobacteria sequences were assigned to the Alphaproteobacteria (51% Type 1; 42% Type 3), specifically the Rhizobiales (11.1% Type 1; 11.3% Type 3), Rhodobacterales (10.1% Type 1; 10.3% Type 3), and Rhodospiralles (8.8% Type 1; 8.7% Type 3). Most, however, of the Alphaproteobacteria protein-encoding gene sequences were unable to be classified beyond the phyla-level in both Type 1 (45.8%) and Type 3 (44.6%) mats. Deltaproteobacteria were also in high abundance with 9.9% of the Type 1 and 13% of the Type 3 proteobacterial sequences. The difference in deltaproteobacterial sequences between mat types was the result of an increase in the number of recovered reads in the Type 3 mats associated with the order Deltasulfobacterales, a taxa predominately composed of sulfate reducing bacteria. Numerous protein-coding reads were also assigned to the Bacteroidetes/Chlorobi (8.6% Type 1; 8.5% Type 3), Firmicutes (3.6% Type 1; 4.4% Type 3), and Actinobacteria (1.6% Type 1 and 3), although no significant differences between mat types were observed at the phyla or class-level (supplemental Figure S1). Pyrosequencing of the metagenome revealed genes assigned to members of eight additional phyla not previously detected in Bahamian stromatolites with 16S rRNA analysis and include: Aquificae, Deferribacteres, Dictyoglomi, Elusimicrobia, Fusobacteria, Synergistetes, Tenericutes, and Thermotogae. The number of assigned reads, however, within all of these phyla was <1% of the sequenced metagenome in both mat types. In addition to the overall taxonomy of the stromatolitic mats, analysis of the metagenomes also provided insight into the environments where similar sequences have been recovered (Figure 3). Most of the recovered sequences were derived from organisms attributed to aquatic, mesophilic (i.e., salinity and temperature) habitats. The range of oxygen tolerance was also examined, 36.2% of recovered genes share similarity to aerobic organisms, 18.4% to facultative anaerobes, and 15.2% obligate anaerobes.

### Comparison of functional genes of stromatolitic mat types

**Figure 4:**
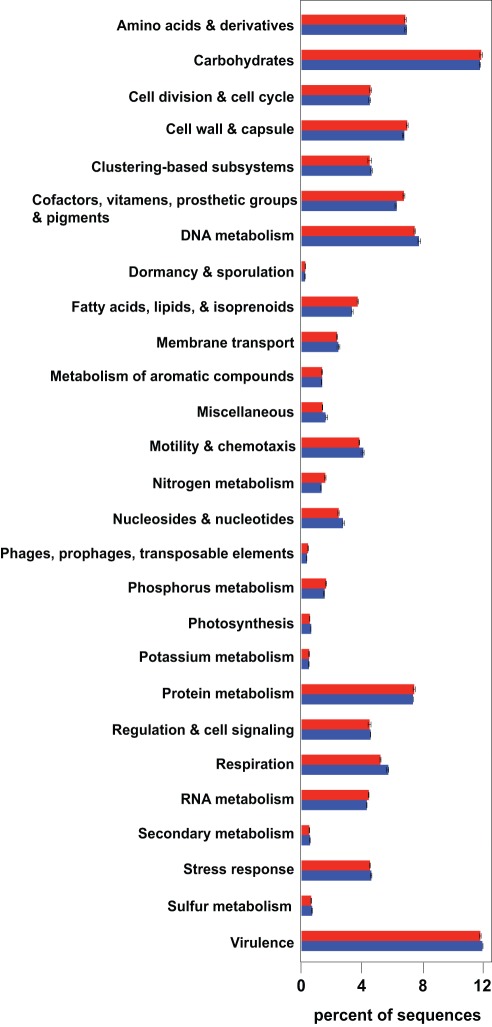
Functional assignment of metagenomic sequences. Percentage of sequences assigned to each functional subsystem using SEED annotation for nonlithifying Type 1 (red) and lithifying Type 3 (blue) stromatolitic mats. Error bars reflect standard error of the mean in the subsystem annotations between the replicate metagenome analyses.

To examine the overall functional gene complexity of the nonlithifying and lithifying stromatolitic mats an equalized number (n = 47,520) of pyrosequences were randomly selected in triplicate and compared to the SEED database [20] using the MetaGenome Rapid Annotation of Sequence Technology (MG-RAST) [21] and the Kyoto Encyclopedia of Genes and Genomes (KEGG) database [22] using BLASTX. The datasets were also statistically compared using XIPE-TOTEC [23] to independently assess differences within the two mat types. Only one third of the recovered sequences (31% Type 1 and 3) were assigned to one of 27 SEED subsystems (Figure 4), with 69% of the sequences unknown in both mats. Subsystem-level analyses of the annotated reads from the stromatolitic mat metagenomes indicated the two dominant subsystems in both mat types were Carbohydrates and Virulence. Statistical analysis of the SEED results using XIPE-TOTEC confirmed the results depicted in Figure 4. There was a statistically significant overrepresentation of the subsystems Cell Wall and Capsule and Protein Metabolism in Type 1 mats and an overrepresentation of subsystems in lithifying Type 3 mats associated with Virulence, Motility and Chemotaxis, Respiration, and Regulation and Cell Signaling. At higher resolution using SEED few differences were observed between the nonlithifying and lithifying stromatolitic mat metagenomes. However, when sequences were compared to the KEGG database and assigned to a KEGG orthology (KO) group additional differences between mat types were observed, which were also confirmed using XIPE-TOTEC. For example in the Carbohydrate Metabolism category (Table 1) differences between nonlithifying and lithifying metagenomes occurred in several pathways associated with organic carbon utilization. The lithifying Type 3 mats had an increase in the number of reads associated with fructose and mannose (ko00051), galactose (ko00052); starch and sucrose (ko00500); and glyocylate and dicarboxylate (ko00660) metabolisms. Other KEGG categories previously shown to be important in stromatolitic mat metabolisms [13] showed few differences between the nonlithifying and lithifying mat metagenomes. For example, in the category Energy Metabolism the only difference in relative abundance was in genes assigned to the photosynthesis pathway (ko00195). Other metabolisms such as carbon fixation (ko00710), nitrogen (ko00910), and sulfur (ko00920) metabolism showed no significant differences in the relative abundance of genes associated with each pathway in Type 1 and Type 3 mats (Table 1). Another functional category shown to be important in stromatolite development is Glycan Biosynthesis and Metabolism. Many of these gene products contribute to the formation of exopolymeric substances, a critical component for the stabilization and accretion of stromatolitic mats. The comparison of metagenomes showed few differences in this category. There were, however, a higher number of genes matching to the peptidoglycan biosynthesis (ko0550) pathway in nonlithifying Type 1 mats, whereas the lithifying Type 3 mats had more sequences with similarity to the glycosylphosphatidylinositol anchor biosynthesis (ko0563) and the glycosphingolipid biosynthesis-globo (ko0603) and ganglio (ko0604) series.

**Table 1:** Comparison of microbialitic mat sequences that share homology to genes in KEGG pathways^a^.

KEGG Class ID	KEGG Category [KEGG orthology (ko): number]	Type 1 Matches^b ^(%)^c^	Type 1 SEM^d^	Type 3 Matches (%)	Type 3 SEM	P-Value^e^
**1100**	**Carbohydrate metabolism**	**1753(14.42)**	**8.57**	**1858 (14.82)**	**15.19**	**0.008**
10	Glycolysis/Gluconeogenesis [PATH:ko00010]	318 (2.62)	0.58	319 (2.55)	6.64	0.860
20	Citrate (TCA cycle)[PATH:ko00020]	198 (1.63)	5.18	211 (1.68)	4.04	0.130
30	Pentose phosphate pathway [PATH:ko00030]	176 (1.45)	3.38	157 (1.25)	4.37	0.025
40	Pentose and glucuronate interconversions [PATH:ko00040]	80 (0.66)	2.60	67 (0.54)	2.60	0.029
51	Fructose and mannose metabolism [PATH:ko00051]	258 (2.12)	4.16	274 (2.18)	1.67	0.049
52	Galactose metabolism [PATH:ko00052]	123 (1.01)	4.04	154 (1.23)	2.52	0.005
53	Ascorbate and aldarate metabolism [PATH:ko00053]	51 (0.42)	1.76	43 (0.34)	2.53	0.137
500	Starch and sucrose metabolism [PATH:ko00500]	293 (2.41)	1.76	316 (2.52)	3.76	0.013
520	Amino sugar and nucleotide sugar metabolism [PATH:ko00520]	346 (2.84)	8.69	349 (2.79)	5.78	0.746
562	Inositol phosphate metabolism [PATH:ko00562]	51 (0.42)	2.89	91 (0.72)	3.33	0.001
620	Pyruvate metabolism [PATH:ko00620]	323 (2.65)	7.17	335 (2.67)	9.28	0.367
630	Glyoxylate and dicarboxylate metabolism [PATH:ko00630]	115 (0.94)	4.48	135 (1.08)	0.67	0.040
640	Propoanate metabolism [PATH:ko00640]	190 (1.56)	2.08	158 (1.26)	4.37	0.008
650	Butanoate metabolism[PATH:ko00650]	194 (1.60)	3.18	192 (1.53)	7.55	0.796
660	C5-Branched dibasic acid metabolism [PATH:ko00660]	34 (0.28)	0.67	40 (0.32)	0.67	0.003
**1120**	**Energy Metabolism**	**1275 (10.49)**	**22.23**	**1326 (10.57)**	**22.53**	**0.189**
190	Oxidative phosphorylation [PATH:ko00190]	462 (3.80)	13.92	482 (3.84)	8.50	0.294
195	Photosynthesis [PATH:ko00195]	120 (0.99)	2.91	142 (3.84)	1.45	0.007
196	Photosynthesis-antenna proteins [PATH:ko00196]	30 (0.24)	1.76	29 (0.23)	2.08	0.819
680	Methane Metabolism [PATH:ko00680]	293 (2.41)	1.76	304 (2.42)	6.36	0.242
710	Carbon fixation in photosynthetic organisms [PATH:ko00710]	159 (1.31)	6.43	180 (0.23)	6.57	0.081
720	Reductive carboxylate cycle (CO2 Fixation) [PATH:ko00720]	153 (1.26)	4.04	151 (1.21)	3.33	0.767
910	Nitrogen metabolism [PATH:ko00910]	182 (1.50)	3.33	185 (1.48)	5.13	0.689
920	Sulfur Metabolism [PATH:ko00920]	68 (0.56)	2.96	61 (0.49)	1.53	0.140
**1170**	**Glycan Biosynthesis and Metabolism** **[PATH:ko1170]**	**518 (4.26)**	**0.88**	**537 (4.28)**	**5.03**	**0.058**
510	N-Glycan biosynthesis [PATH:ko0510]	63 (0.52)	1.20	63 (0.50)	2.91	1.000
511	Other glycan degradation [PATH:ko0511]	34 (0.28)	1.20	57 (0.46)	2.19	0.002
512	High-mannose type N-glycan biosynthesis [PATH:ko0512]	6 (0.05)	0.58	5 (0.04)	0.33	0.134
513	O-Mannosyl glycan biosynthesis [PATH:ko0513]	2 (0.02)	0.58	4 (0.03)	0.58	0.070
514	O-Glycan biosynthesis [PATH:ko0514]	3 (0.02)	0.58	8 (0.06)	0.58	0.004
531	Glycosaminoglycan degradation [PATH:ko0531]	32 (0.26)	3.51	40 (0.32)	2.03	0.126
532	Glycosaminoglycan biosynthesis-chondroitin sulfate [PATH:ko0532]	7 (0.05)	0.88	6 (0.05)	1.15	0.672
533	Glycosaminoglycan biosynthesis-keratan sulfate [PATH:ko0533]	1 (0.01)	0.58	3 (0.03)	0.67	0.058
534	Glycosaminoglycan biosynthesis-heparan sulfate [PATH:ko0534]	10 (0.08)	0.58	15 (0.12)	1.86	0.118
540	Lipopolysaccharide biosynthesis [PATH:ko0540]	92 (0.76)	4.05	97 (0.78)	1.20	0.343
550	Peptidoglycan biosynthesis [PATH:ko0550]	261 (2.15)	3.46	227 (1.81)	0.88	0.007
563	Glycosylphosphatidylinositol anchor biosynthesis [PATH:ko0563]	15 (0.12)	2.19	23 (0.19)	1.20	0.038
601	Glycosphingolipid biosynthesis-lacto & neolacto [PATH:ko0601]	3 (0.02)	0.33	5 (0.04)	0.88	0.139
603	Glycosphingolipid biosynthesis-globo series [PATH:ko0603]	13 (0.10)	0.88	17 (0.13)	0.67	0.025
604	Glycosphingolipid biosynthesis-ganglio series [PATH:ko0604]	7 (0.06)	1.53	13 (0.11)	0.67	0.038

a pyrosequencing reads were compared to KEGG database using a cutoff e-value of 10^−5^.

b number of matches reflect the mean of three replicate MEGAN analyses.

c percent of reads found within in each category.

d standard error of the mean calculated for three replicates.

e p-values reflect result of two-tailed t-test between microbialitic mat types.

### Substrate utilization patterns of stromatolitic mat types

**Figure 5:**
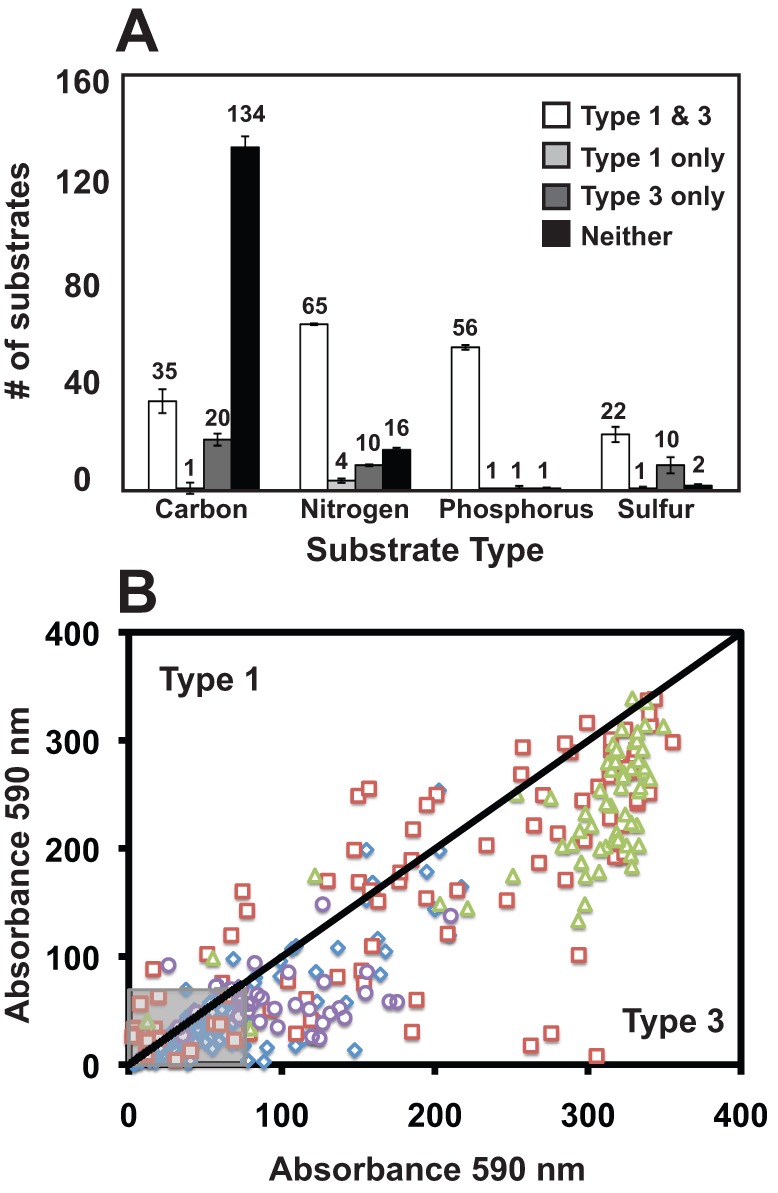
Overview of substrate utilization patterns in stromatolitic mats using phenotypic microarrays. A. The specific number of carbon, nitrogen, phosphorus and sulfur substrates used by the Type 1 and 3 communities are listed at the top of each column. Error bars reflect standard error of the mean between three independent replicates of the microarray assays using microbial mat slurries. B. Comparison of absorbance readings between Type 1 and 3 mats indicating higher utilization of most carbon (blue diamonds), nitrogen (red squares), phosphorus (green triangles) and sulfur (purple circles) substrates by lithifying Type 3 mats. Grey box represents those substrates below threshold absorbance levels.

**Figure 6:**
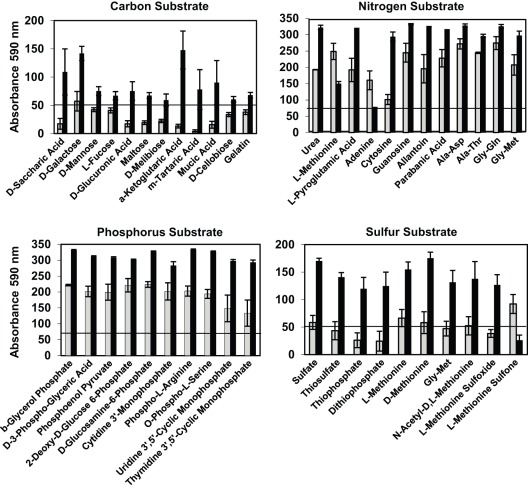
Comparison of substrate utilization patterns in stromatolitic mats. Selected examples of carbon, nitrogen, phosphorus, and sulfur substrate utilization in nonlithifying Type 1 (gray) and lithifying Type 3 (black) stromatolitic mats. The horizontal line at 50–70 U denotes the background level.

To examine the metabolic activity of the nonlithifying and lithifying mat communities live mat samples were analyzed with metabolic phenotypic microarrays with a wide variety of carbon (C), nitrogen (N), phosphorus (P) and sulfur (S) substrates. Slurries of nonlithifying and lithifying mats were incubated with the microarray plates in triplicate for 48 h under aerobic conditions (for details please refer to Materials and Methods). A summary of the overall utilization patterns is visualized in Figure 5 and examples of specific substrates are shown in Figure 6. A full list of substrates and their utilization are listed in supplemental Tables S1, S2, S3, and S4. A total of 18% of the C substrates (n = 35) were metabolized by both mat types and were primarily carboxylic acids, mono- and disaccharides (Figure 6; Table S1). Although both mats types were capable of using the 35 substrates (Figure 5A), the extent of utilization by the mat types differed in most of the substrates and was higher in Type 3 mats (Figure 5B). The only C substrate found to be exclusively used in Type 1 mats was fumaric acid. A higher number of C substrates (n = 20) were utilized by organisms in the lithifying Type 3 mats, such as L-fucose, D-mannose, D-glucuronic acid, and D-trehalose (Figure 6; Table S1). The majority of C substrates tested, however, were unable to be utilized by the Type 1 and 3 mat communities under the experimental testing conditions (Figure 5; Table S1).

Overall utilization of N, P, S substrates was higher compared to the C sources (Figure 5B). Of the 95 tested N substrates 68% (n = 65) were metabolized by both Type 1 and 3 mats by 48 h (Figure 5). These N substrates included amino acid dipeptides with L-alanine or glycine at the amino terminus and cyclic compounds with an available amino group (Table S2). As with the C substrates Type 3 mats were able to more strongly utilize the N substrates (Figure 5B) with the exception of L-methionine and adenine (Figure 6). Type 3 mats also exclusively utilized 11% of substrates, compared to only 4% in Type 1 mats, and included amines with a terminal nitrogen and a few nucleosides (Table S2). When grown on various P substrates, both mat types utilized all but three of the 59 substrates with Type 3 mats having a higher utilization rate (Figure 5B) particularly in those substrates associated with purine cyclic and pyrimidine monophosphates (Figure 6). One of the tested P substrates exclusive to Type 1 mats was hypophosphite, while triethyl phosphate, was specific to Type 3 mats. Lastly, of the 35 tested S substrates, 63% (n = 22) were utilized by both mat types and included derivatives of cysteine and various sulfonic acids. Although both mats utilized sulfate, there was a 3-fold increase in the extent of sulfate metabolism in the Type 3 mats (Figure 6). Type 3 mats also strongly utilized an additional 10 substrates such as thiophosphates and methionine compounds. Type 1 mats exclusively utilized only one substrate, L-methionine sulfone, after 48 h of incubation (Table S4).

### Linking the metagenome to the metabolic activity of the stromatolitic mats

**Figure 7:**
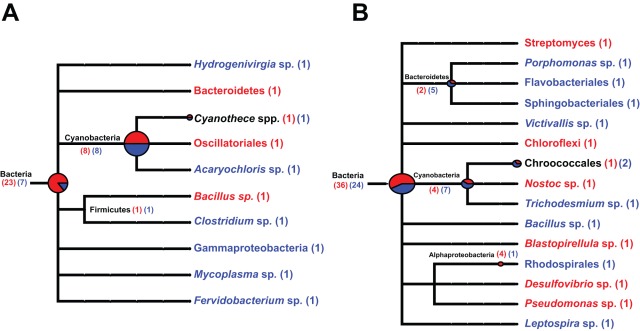
Screening of metagenomes for specific carbohydrate metabolism using MEGAN. Distribution of taxa that harbor genes associated with galactose (A) and mannose (B) utilization. Most of the recovered functional genes associated with galactose and mannose utilization are unable to be assigned beyond domain and phyla level. The number of genes recovered from Type 1 (red) and Type 3 (blue) mat types that could be assigned to taxa are listed in parentheses.

Once substrates were identified as being differentially utilized by the mat types, the mat metagenomes were then screened to delineate the potential organisms associated with these metabolic activities. For example, of the various carbon substrates metabolized by the stromatolitic communities, D-galactose and D-mannose had pronounced differences in the extent of utilization between the two mat types (Figure 6). Screening of the mat metagenomes for all genes associated with galactose and mannose utilization enabled the taxonomic identification of some of the organisms within the Type 1 and 3 mat communities that harbor these gene pathways (Figure 7). MEGAN analysis of the metagenome indicated more than half of the recovered galactose genes were putatively derived from Cyanobacteria (Figure 7A). In mannose utilization, only 28% of the recovered genes were assignable to taxa. Of those assigned sequences that were identified, 18% were attributed to Cyanobacteria, 13% to the Proteobacteria, and 12% to the Bacteroidetes (Figure 7B).

## Discussion

### Microbial community in the stromatolitic mats

Taxonomic analysis of stromatolitic mat metagenomes confirmed previous SSU rRNA analyses that the stromatolite communities are predominately bacterial [8]. More than half of the recovered pyrosequences shared similarity to protein-encoding genes assigned to Bacteria and together resembled the taxa associated with the metagenomes of other lithifying microbial mats, such as the fresh water microbialites of Cuatro Ciénegas, Mexico [19]. Analysis of the functional genes within the stromatolites also confirmed the dominance of Cyanobacteria and Proteobacteria within the mat communities. Cyanobacteria are considered the driving metabolic force within the stromatolitic community and essential for carbonate deposition [13], [24], [25]. More than a quarter of the recovered bacterial reads were derived from Cyanobacteria, specifically the Chroococcales, Oscillatoriales and Nostocales. With the exception of the Nostocales, both the Chroococcales and the Oscillatoriales have been well documented in lithifying microbial mats from a wide range of environmental habitats including freshwater, marine, and hypersaline conditions [8], [19], [26]. The detection of the heterocystous cyanobacterial genes in the stromatolite metagenome is new and may reflect the absence of deep sequencing in the previous 16S rRNA gene studies or may simply be an over representation in the recovered sequences due to the larger genome size (∼5–6 Mb) and increased number of sequenced Nostocales genomes in the NCBI-nr database. Regardless, the presence of predominantly diazotrophic cyanobacteria (>70%), such as *Cyanothece* and *Synechococcus*, within the metagenomes of both mat types is supported by previous biochemical analysis demonstrating nitrogenous activity and numerous recovered dinitrogenase reductase (*nifH*) genes from Chroococcales [27]. Screening of the stromatolitic mat metagenomes also revealed additional Chroococcales-like genes associated with nitrogen fixation, such as scaffold assembly proteins (*nifE*), cofactor carrier proteins (*nifX*), stabilizing proteins (*nifW*) and nitrogenase-specific transcriptional regulators.

The metagenomic sequencing also provided the first insight into the eukaryotic community of the Bahamian stromatolites. Although eukaryotes comprised only 7% of the Type 1 mat metagenome and 13% of the Type 3 metagenome, the diversity of recovered sequences was high (Figure 2A). Both stromatolitic mat types contained a high number of mixotrophic protists, such as Alveolata, Amoebozoa, Cryptophyta and Euglenozoa. Protists are considered to be the main consumers of bacteria and have been shown to influence the community structure of microbial communities through selective grazing [28], [29]. For example, the cryptophyte *Goniomonas* has been shown to selectively graze on Gammaproteobacteria [30] and several other phagotrophic protists, such as Alveolata and stramenophiles, selectively targeted coccoid cyanobacteria [31]. In addition to the protists, metazoans such as nematodes (Pseudocoelomata) were also found in high abundance in both mat types (Figure 2). Previous studies have shown that nematodes are enriched in other lithifying microbial mat communities, such as the unlaminated thrombolites also located at Highborne Cay [32]. Much like the phagotrophic protists, nematodes are active grazers of microbes and have been shown to be attracted to volatile organic compounds generated by cyanobacteria-dominated biofilms [33]. These results suggest that the eukaryotic population may play a role in controlling the bacterial composition of the stromatolitic mats and possibly contribute to nutrient cycling within the mats, as phagocytosis has been shown to be a critical process in the regeneration of inorganic nutrients [34], [35]. The only pronounced difference in the eukaryotic populations between the mat types was the increase in Viridiplantae in the lithifying Type 3 mat type. Viridiplantae are found associated with a wide range of microbial mat communities including freshwater microbialites [19], [36]. The 10-fold increase in recovered sequences in Type 3 mats suggests an opportunistic colonization of the surfaces of the Type 3 mats, as these mats often form during long periods of exposure from sand burial [10]. Previous studies have shown that photosynthetic eukaryotes on the surfaces of stromatolitic mats do not recover from extended sand burial events [37], which have been shown to be a critical trigger for the formation of nonlithifying Type 1 mats and may indicate why Viridiplantae-like organisms are not as abundant in the Type 1 mat communities [10].

### Functional complexity in stromatolitic mats

Together, the taxonomic complexity of the stromatolitic mat communities results in a broad range of metabolic processes that are highly interdependent with regard to energy metabolism, nutrient cycling, and the mechanisms underlying carbonate precipitation [13]–[15]. Previous biogeochemical analyses of the stromatolitic mats have identified steep vertical gradients of key geochemical indicators (e.g. oxygen, sulfide, pH) that result in pronounced microenvironments [13]. Within these microenvironments coupled reduction and oxidation reactions via elemental cycling (e.g. carbon, nitrogen, phosphorus, sulfur) support the formation of robust biogeochemical cycles [5], [15]. The preferential utilization of a certain biogeochemical cycle or metabolic pathway over another can influence the extent of lithification within the microbial mats [15]. For example, oxygenic and anoxygenic photosynthesis, as well as sulfate reduction, are known to increase the alkalinity of the surrounding microenvironment thus promoting carbonate precipitation [5], [14], [38], [39]. Contrastingly, metabolisms such as aerobic respiration, sulfide oxidation, and fermentation are more likely to induce mineral dissolution [13]. Analysis of both the nonlithifying and lithifying stromatolitic mat metagenomes have identified numerous genes associated with all of these aforementioned metabolic pathways (Figure 2, Table 1) suggesting that both mat types have the potential for mineralization. With the exception of oxygenic photosynthesis the relative abundance of genes associated with these key metabolisms was not statistically different between mat types (Table 1). The statistical increase in genes in oxygenic photosynthesis in lithifying Type 3 mats may be directly correlated to the increase in eukaryotic phototrophs (e.g. Viridiplantae) detected in the lithifying mat metagenome and may play a role in increasing alkalinity and shifting carbonate equilibrium towards precipitation. Although both mat types possessed genes associated with the full range of metabolisms associated with carbonate regulation, the expression and utilization of these genes are likely to be spatially and temporally differentially regulated between the two mat types, as has been shown in many other marine environments [40]–[42].

Despite the few statistically significant differences observed between mat types in Energy Metabolism, there were differences associated with the functional gene category Carbohydrate Metabolism (Table 1). In the lithifying Type 3 mats there was a pronounced increase in the number of genes associated with the metabolism of carbohydrate monomers such as galactose and mannose metabolism. These metagenomic differences were complemented by the metabolic phenotypic microarray analysis, which revealed a pronounced increase in substrate diversity and utilization within the lithified Type 3 mats. Utilization of key hexoses (D-galactose, D-mannose), pentoses (D-arabinose), deoxy sugars (L-fucose), and acidic sugars (D-glucaronic acid, D-galacturonic acid) were higher in Type 3 mats (Figure 6).

Together these results suggest that the microbial community within the Type 3 mats may have additional pathways and/or a higher propensity to degrade exopolymeric substances (EPS). EPS materials plays an important role in the carbonate formation within the stromatolites and are predominantly produced by cyanobacteria [43], [44] and sulfate-reducing bacteria [45]. Cyanobacterial EPS derived from Bahamian stromatolitic mats have been shown to contain approximately 50% carbohydrate, consisting primarily of glucose, galactose, xylose, and fucose with the remaining material comprised of proteins, uronic acids, and glucosamine glycans [46]. The abundance of negatively charged acidic functional groups (e.g. carboxylic acids and sulfate) within the EPS material has been shown to increase the binding of mono- and divalent cations (e.g. Ca^2+^), thus removing free ions from the surrounding environment and in effect inhibiting carbonate precipitation [15], [47]. Other compounds such as acidic amino acids and uronic acids have also been shown to be inhibitors of calcium carbonate precipitation [9]. Through the microbial degradation and reorganization of the EPS material, previous studies have shown that the Ca-binding capacity of the EPS material can be reduced [14]. The increase in the relative abundance of genes associated with the heterotrophic degradation of hexoses (e.g. D-galactose, D-mannose) and dicarboxylic acids in lithified Type 3 mats (Table 1) may suggest an increased metabolic capacity of this mat type for the microbial degradation of EPS and the release of Ca^2+^. The liberated Ca^2+^ could then potentially serve as a nucleation site with the EPS matrix, thus facilitating the precipitation of carbonate in the Type 3 mats.

Lastly, the increased utilization of low molecular weight organic acids may be indicative of elevated sulfate reduction in the Type 3 mats. Sulfate reduction has been shown to be a dominant metabolism in stromatolitic mats [48], [49]. In previous studies lithifying mat slurries incubated with cyanobacterial EPS, sugars and sulfonates exhibited a significant increase in sulfate reduction, as well as the degradation of these substrates under both oxic and anoxic conditions [48]. Therefore, the pronounced increase in the utilization of sulfate (3-fold) and other sulfur substrates in the Type 3 mats (Table S4) may suggest an increase of sulfate reduction in this mat type. Sulfate reduction has been shown to increase the alkalinity of the surrounding environment through the metabolism of sulfate and production of sulfide [50]–[53]. The resulting increase in alkalinity coupled with free Ca^2+^ ions due to microbial degradation of EPS may be driving carbonate equilibrium towards lithification in the Type 3 mats.

### Conclusions

In summary, we have profiled the underlying molecular pathways and processes associated with the nonlithifying and lithifying stromatolitic mats of Highborne Cay, The Bahamas. Metagenomic analyses of the stromatolitic mats revealed that lithifying Type 3 stromatolitic mats had an increased relative abundance of genes associated with the metabolism of carbohydrates known to be constituents of the EPS matrix of stromatolites. This increase in gene abundance was correlated to an increase in organic carbon utilization by the lithifying mats, providing a strong link between the metagenome and the physiology within the stromatolitic mat communities. The study also enabled associations to be made between specific microbial taxa with metabolic activities in the mats (Figure 7). By screening the metagenomes for genes of interest and correlating those genes to various taxa, it is now possible to assess which microbes are associated with those metabolisms linked to stromatolite accretion and development. Although this work provides a framework for elucidating the metabolic potential of these ecosystems, future sequencing of the stromatolitic mat metatranscriptomes will be required to characterize the expression of these targeted genetic pathways over spatial and temporal scales, further delineating the molecular mechanisms that regulate carbonate mineralization and the formation of stromatolites.

## Materials and Methods

### Stromatolitic mat sample collection

All stromatolitic mat samples were collected from the island of Highborne Cay located in the Exuma Sound, The Bahamas in November 2009. Nonlithifying mats (Type 1) were collected from Site 2, whereas lithifying stromatolitic mats (Type 3) were collected at Site 10, approximately 500 m from each other. Site designations are based on Andres et al., [54]. The temperature (24°C), salinity (38‰) and surface photosynthetic active radiation 2200 µE/m^2^/s (12:30 p.m.) were identical for both locations. The water depth varied extensively throughout the day for both subtidal sites and was due to the high wave action of these near-shore stromatolitic mats. The water chemistry was homogenous throughout all ten collection sites (Pieter Visscher, pers. comm.). Live samples for substrate utilization profiling were transported to the Space Life Science Lab at the Kennedy Space Center, FL where they were incubated in seawater at 24°C at 2000 µE/m^2^/s for 48 h until processing. All necessary collection permits were obtained for the described field studies from the Bahamian Ministry of Agriculture and Marine Resources.

### DNA extraction and sequencing

DNA was extracted from the upper 8 mm of the frozen mat samples as previously described [11], [55]. In both stromatolitic mat types the upper 8 mm represent the accreting living mat and include: 1) surface EPS-rich layer (0–0.5 mm); 2) oxic layer (0.5–5 mm) and, 3) lower anaerobic layer (5–8 mm) [5], [15]. The differences between the mat types were in the presence of a micritic crust on the surface and fused grain layer in the oxic zone layer of Type 3 mats, as visualized in Figure 1. Briefly, vertical sections (100 mg) that contained all three layers were incubated in an extraction buffer that contained 100 mM Tris-HCl pH 8.0, 100 mM EDTA, 1% (w/v) cetyl trimethyl ammonium bromide, 2% (w/v) sodium dodecyl sulfate, and a cocktail of sterile glass beads (0.2 g 0.1 mm; 0.2 g 0.7 mm; and eight 2.4 mm; Biospec, Bartlesville, OK). The samples were bead-beat for 2 min then a concentrated xanthogenate solution was added, which contained 2.5 M ammonium acetate and 3.2% (w/v) potassium ethyl xanthogenate. The samples were then incubated at 65^°^C for 2 h, placed on ice for 30 min and centrifuged. The supernatant containing the DNA was mixed with a KCl solution such that the final concentration was 0.5 M KCl and then centrifuged. The recovered supernatant was mixed with 5 M NaCl and 2 volumes of cold 100% ethanol and stored overnight at −80°C. DNA was recovered through centrifugation and the pellets were air dried before resuspension in C4 solution (MoBio PowerSoil DNA kit, MoBio, Carlsbad, CA). The DNA was recovered using the remaining MoBio Power Soil kit reagents according to manufacturer’s instructions. Concentrations of genomic DNA were determined with Quant-iT PicoGreen ds DNA Assay Kit (Invitrogen, Molecular Probes, Eugene, OR) and quality was determined spectrophotometrically with the NanoDrop 1000 (ThermoScientific, Waltham, MA). Replicate extractions (n = 3) were normalized and pooled. Recovered genomic DNA (1.5 µg per mat type) was sequenced using a 454 GS-FLX pyrosequencer with Titanium chemistry (Roche, Indianapolis, IN) at the University of Florida Interdisciplinary Center for Biotechnology Research (Gainesville, FL).

### Analysis of metagenomic sequencing data

To identify and remove potential artifacts in the recovered 454 sequencing reads, the metagenomic libraries were pre-processed and screened for ambiguous reads and artificial replicated sequences using the method described in Gomez-Alvarez et al., [56] with 9.59% and 8.79% of sequences from the nonlithifying (Type 1) and lithifying (Type 3) mats removed, respectively. The remaining high quality reads were then equalized using a random sequence selector PERL script, which selects 75% (n = 47,520) of the total number of quality reads of the smaller data set (Paul Stothard, www.ualberta.ca/stothard/software.html). Three replicate equalized data sets were generated and individually compared to the NCBI-nr database using BLASTX [57]. The resulting alignments were examined with MEGAN 4.0 [58], which uses an algorithm to assign each read to the lowest common ancestor (LCA) of the closest related taxa using NCBI nomenclature. The LCA algorithm parameters, for all alignments, included a bit score of 35 and retained only those reads within 10% of the best hit. The data sets were also examined using the non-parametric statistical analysis program XIPE-TOTEC [23] to assess whether there were differences detected in the two mat populations. Both SEED and KEGG data were compared at using the same sample size (500,000) at 95% confidence. The metagenomic libraries were also annotated using the MetaGenomic Rapid Annotation using Subsystem Technology (MG-RAST) server [59] with the parameters bp>50, E>0.00001 [21]. The metagenomic data sets are publically available through the MG-RAST website under the project names “Stromatolite Type 1 – HBC” (ID 4449591.3) and “Stromatolite Type 3 – HBC” (ID 4449590.3). The raw sequence reads and quality files were deposited into the GenBank NCBI short read archive under accession numbers SRA048308.1 and SRA048309.1.

### Metabolic phenotypic microarrays

Slurries for each mat type were generated by placing 500 mg of freshly collected mat material into 2 ml of filter-sterilized seawater. The samples were then vortexed for 15 min to break up the mat material and dislodge the sand grains from the stromatolitic mats. The mats were then centrifuged at low speeds to only remove the sand grains. Optical densities were determined spectrophotometrically (Genesys 20, Thermo Fisher Scientific, Waltham, MA) at 590 nm absorbance and normalized with filter-sterilized seawater. Phenotype Microarray (PM) plates (Biolog Inc., Hayward, CA) were used to screen the metabolic capability of the mat types. The PM plates contained a variety of individual substrates including carbon (PM1, PM2A), nitrogen (PM3B), phosphorus and sulfur (PM4A) and were inoculated with aliquots (100 µl) of diluted mat slurries. Nitrogen, phosphorus and sulfur plates were supplemented with a carbon source solution of 2 µM ferric citrate as this carbon source was utilized equally by both Type 1 and 3 mat types. All plates were incubated at 30^°^C, for up to 48 h and screened with an Omnilog reader at an absorbance of 590 nm every 15 min (Biolog, Inc., Hayward, CA). Absorbance readings taken at 24 and 48 h were analyzed with the parametric software (v1.3) package of the Omnilog reader (Biolog Inc. Hayward, CA). A substrate was considered utilized by the community if the absorbance reading was above the threshold level. The threshold was set at 20% of the highest absorbance detected on each plate. The resulting replicate utilization patterns between mat types was compared using a student’s T-test and considered significant if p≤0.05.

## Supporting Information

Figure S1 **Bacterial composition of the stromatolite metagenomes at the class-level using MEGAN analysis.** Pyrosequencing reads assigned to the bacterial classes. Reads derived from nonlithifying Type 1 mats are in red, whereas reads from lithifying Type 3 mats are in blue. The relative abundance of reads associated with each taxa are listed in parentheses, with Type 1 and 3 mats listed, respectively.
(EPS)

Table S1 **Carbon substrate absorbance units of stromatolitic microbial mats.** Substrates were considered utilized if absorbance readings were above threshold of 50 units. Values represent mean absorbance unit for three replicate phenotypic microarrays.
(DOCX)

Table S2 **Nitrogen substrate absorbance units of stromatolitic microbial mats.** Substrates were considered utilized if absorbance readings were above threshold of 50 units. Values represent mean absorbance unit for three replicate phenotypic microarrays.
(DOCX)

Table S3 **Phosphorus substrate absorbance units of stromatolitic microbial mats.** Substrates were considered utilized if absorbance readings were above threshold of 50 units. Values represent mean absorbance unit for three replicate phenotypic microarrays.
(DOCX)

Table S4 **Sulfur substrate absorbance units of stromatolitic microbial mats.** Substrates were considered utilized if absorbance readings were above threshold of 50 units. Values represent mean absorbance unit for three replicate phenotypic microarrays.
(DOCX)

## References

[1] Walter MR, Bengston S (1994). Stromatolites: the main geological source of information on the evolution of the early benthos.. Early Life on Earth Nobel Symposium: Columbia University Press.

[2] Grotzinger JP, Knoll AH (1999). Stromatolites in Precambrian carbonates: evolutionary mileposts or environmental dipsticks?. Annual Review of Earth and Planetary Sciences.

[3] Visscher PT, Reid RP, Bebout BM, Hoeft SE, Macintyre IG (1998). Formation of lithified micritic laminae in modern marine stromatolites (Bahamas): The role of sulfur cycling.. American Mineralogist.

[4] Reid RP, Visscher PT, Decho AW, Stolz JF, Bebout BM (2000). The role of microbes in accretion, lamination and early lithification of modern marine stromatolites.. Nature.

[5] Visscher PT, Reid RP, Bebout BM (2000). Microscale observations of sulfate reduction: correlation of microbial activity with lithified micritic laminae in modern marine stromatolites.. Geology.

[6] Macintyre IG, Prufert-Bebout L, Reid RP (2000). The role of endolithic cyanobacteria in the formation of lithified lamiae in Bahamian stromatolites.. Sedimentology.

[7] Stolz JF, Reid RP, Visscher PT, Decho AW, Norman RS (2009). The microbial communities of the modern marine stromatolites at Highborne Cay, Bahamas.. Atoll Research Bulletin.

[8] Baumgartner LK, Spear JR, Buckley DH, Pace NR, Reid RP (2009). Microbial diversity in modern marine stromatolites, Highborne Cay, Bahamas.. Environmental Microbiology.

[9] Kawaguchi T, Decho AW (2002). Isolation and biochemical characterization of extracellular polymeric secretions (EPS) from modern soft marine stromatolites (Bahamas) and its inhibitory effect on CaCO_3_ precipitation.. Preparative Biochemistry and Biotechnology.

[10] Bowlin EM, Klaus J, Foster JS, Andres M, Custals L (2011). Environmental controls on microbial community cycling in modern marine stromatolites.. http://dx.doi.org/10.1016/j.sedgeo.2011.1008.1002.

[11] Foster JS, Green SJ, Ahrendt SR, Hetherington KL, Golubic S (2009). Molecular and morphological characterization of cyanobacterial diversity in the marine stromatolites of Highborne Cay, Bahamas.. ISME Journal.

[12] Foster JS, Green SJ, Seckbach J, Tewari V (2011). Microbial diversity in modern stromatolites. Cellular Origin, Life in Extreme Habitats and Astrobiology: Interactions with Sediments: Springer.

[13] Visscher PT, Stolz JF (2005). Microbial mats as bioreactors: populations, processes and products.. Palaeogeography, Palaeoclimatology, Palaeoecology.

[14] Dupraz C, Visscher PT (2005). Microbial lithification in marine stromatolites and hypersaline mats.. Trends in Microbiology.

[15] Dupraz C, Reid RP, Braissant O, Decho AW, Norman RS (2009). Processes of carbonate precipitation in modern microbial mats.. Earth Science Reviews.

[16] Desnues CG, Rodriguez-Brito B, Rayhawk S, Kelley S, Tran T (2008). Biodiversity and biogeography of phages in modern stromatolites and thrombolites.. Nature.

[17] Handelsman J (2004). Metagenomics: application of genomics to uncultured microorganisms.. Microbiology and Molecular Biology Reviews.

[18] Edwards RA, Rodriguez-Brito B, Wegley L, Haynes M, Breitbart M (2006). Using pyrosequencing to shed light on deep mine microbial ecology.. BMC Genomics.

[19] Breitbart M, Hoare A, Nitti A, Siefert J, Haynes M (2009). Metagenomic and stable isotopic analyses of modern freshwater microbialites in Cuatro Ciénegas, Mexico.. Environmental Microbiology.

[20] Overbeek R, Begley T, Butler RM, Choudhuri JV, Chuang HY (2005). The subsystems approach to genome annotation and its use in the project to annotate 1000 genomes.. Nucleic Acids Research.

[21] Meyer F, Paarmann D, D'Souza M, Olson R, Glass EM (2008). The metagenomics RAST server – a public resource for the automatic phylogenetic and functional analysis of metagenomes.. BMC Bioinformatics.

[22] Kanehisa M, Goto S, Kawashima S, Okuno Y, Hattori M (2004). The KEGG resource for deciphering the genome.. Nucleic Acids Research.

[23] Rodriguez-Brito B, Rohwer F, Edwards RA (2006). An application of statistics to comparative metagenomics.. BMC Bioinformatics.

[24] Pinckney JL, Reid RP (1997). Productivity and community composition of stromatolitic microbial mats in the Exuma Cays, Bahamas.. FACIES.

[25] Dupraz C, Visscher PT, Baumgartner LK, Reid RP (2004). Microbe-mineral interactions: early carbonate precipitation in a hypersaline lake (Eleuthera Island, Bahamas).. Sedimentology.

[26] Burns BP, Goh F, Allen M, Neilan BA (2004). Microbial diversity of extant stromatolites in the hypersaline marine environment of Shark Bay, Australia.. Environmental Microbiology.

[27] Steppe TF, Pinckney JL, Dyble J, Paerl HW (2001). Diazotrophy in modern marine Bahamian stromatolites.. Microb Ecol.

[28] Jurgens K, Matz C (2002). Predation as a shaping force for the phenotypic and genotypic composition of planktonic bacteria.. Antonie Van Leeuwenhoek.

[29] Apple JK, Strom SL, Palenik B, Brahamsha B (2011). Variability in protist grazing and growth on different marine *Synechococcus* isolates.. Applied and Environmental Microbiology.

[30] Jezbera J, Hornak K, Simek K (2005). Food selection by bacterivorous protists: insight from the analysis of the food vacuole content by means of fluorescence in situ hybridization.. FEMS Microbiology Ecology.

[31] Frias-Lopez J, Thompson A, Waldbauer J, Chisholm SW (2009). Use of stable isotope-labelled cells to identify active grazers of picocyanobacteria in ocean surface waters.. Environmental Microbiology.

[32] Myshrall K, Mobberley JM, Green SJ, Visscher PT, Havemann SA (2010). Biogeochemical cycling and microbial diversity in the modern marine thrombolites of Highborne Cay, Bahamas.. Geobiology.

[33] Höckelmann C, Moens T, Jüttner F (2004). Odor compounds from cyanobacterial biofilms acting as attractants and repellents for free-living nematodes.. Limnology and Oceanography.

[34] Güde H (1985). Influence of phagotrophic processes on the regeneration of nutrients in two stage continuous culture systems.. Microbial Ecology.

[35] Moreno AM, Matz C, Kjelleberg S, Manefield M (2010). Identification of ciliate grazers of autotrophic bacteria in ammonia-oxidizing activated sludge by RNA stable isotope probing.. Applied and Environmental Microbiology.

[36] Varin T, Lovejoy C, Jungblut AD, Vincent WF, Corbeil J (2010). Metagenomic profiling of Arctic microbial mat communities as nutrient scavenging and recycling systems.. Limnology and Oceanography.

[37] Perkins RG, Kromkamp JC, Reid RP (2007). Importance of light and oxygen for photochemical reactivation in photosynthetic stromatolite communities after natural sand burial.. Marine Ecology-Progress Series.

[38] Decho AW (1990). Microbial exopolymer secretions in ocean environments – their role(s) in food webs and marine processes.. Oceanography and Marine Biology.

[39] Paerl HW, Steppe TF, Reid RP (2001). Bacterially mediated precipitation in marine stromatolites.. Environmental Microbiology.

[40] Hewson I, Poretsky RS, Tripp HJ, Montoya JP, Zehr JP (2010). Spatial patterns and light-driven variation of microbial population gene expression in surface waters of the oligotrophic open ocean.. Environmental Microbiology.

[41] Shi Y, Tyson GW, Eppley JM, DeLong EF (2011). Integrated metatranscriptomic and metagenomic analyses of stratified microbial assemblages in the open ocean.. ISME Journal.

[42] McCarren J, Becker JW, Repeta DJ, Shi Y, Young CR (2010). Microbial community transcriptomes reveal microbes and metabolic pathways associated with dissolved organic matter turnover in the sea.. Proceedings of the National Academy of Sciences.

[43] De Philippis R, Margheri MC, Materassi R, Vincenzini M (1998). Potential of unicellular cyanobacteria from saline environments as exopolysaccharide producers.. Applied and Environmental Microbiology.

[44] Stal LJ (2003). Microphytobenthos, their extracellular polymeric substances, and the morphogenesis of intertidal sediments.. Geomicrobiology Journal.

[45] Braissant O, Decho AW, Dupraz C, Glunk C, Przekop KM (2007). Exopolymeric substances of sulfate-reducing bacteria: interactions with calcium at alkaline pH and implication for formation of carbonate minerals.. Geobiology.

[46] Kawaguchi T, Decho AW (2000). Biochemical characterization of cyanobacterial extracellular polymers (EPS) from modern marine stromatolites (Bahamas).. Preparative Biochemistry and Biotechnology.

[47] Bianchi TS (2007). Biochemistry of Estuaries..

[48] Visscher PT, Gritzer RF, Leadbetter ER (1999). Low-molecular-weight sulfonates, a major substrate for sulfate reducers in marine microbial mats.. Applied and Environmental Microbiology.

[49] Visscher PT, Surgeon TM, Hoeft SE, Bebout BM, Thompson JA, Taillefert M, Rozan T (2002). Microelectrode studies in modern marine stromatolites: unraveling the Earth's past.. Electrochemical methods for the environmental analysis of trace metal biogeochemistry.

[50] Canfield DE, Des Marais DJ (1991). Aerobic sulfate reduction in microbial mats.. Science.

[51] Minz D, Fishbain S, Green SJ, Muyzer G, Cohen Y (1999). Unexpected population distribution in a microbial mat community: sulfate-reducing bacteria localized to the highly oxic chemocline in contrast to a eukaryotic preference for anoxia.. Applied and Environmental Microbiology.

[52] Orphan VJ, Hinrichs KU, Ussler W, Paull CK, Taylor LT (2001). Comparative analysis of methane-oxidizing archaea and sulfate-reducing bacteria in anoxic marine sediments.. Applied and Environmental Microbiology.

[53] Baumgartner LK, Reid RP, Dupraz C, Decho AW, Buckley DH (2006). Sulfate reducing bacteria in microbial mats: changing paradigms, new discoveries.. Sedimentary Geology.

[54] Badger MR, Price GD, Long BM, Woodger FJ (2006). The environmental plasticity and ecological genomics of the cyanobacterial CO_2_ concentrating mechanism.. Journal of Experimental Botany.

[55] Green SJ, Blackford C, Bucki P, Jahnke LL, Prufert-Bebout L (2008). A salinity and sulfate manipulation of hypersaline microbial mats reveals stasis in the cyanobacterial community structure.. ISME Journal.

[56] Gomez-Alvarez V, Teal TK, Schmidt TM (2009). Systematic artifacts in metagenomes from complex microbial communities.. ISME Journal.

[57] Altschul SF, Madden TL, Schaffer AA, Zhang J, Zhang Z (1997). Gapped BLAST and PSI-BLAST: a new generation of protein database search programs.. Nucleic Acids Research.

[58] Huson DH, Auch AF, Qi J, Schuster SC (2007). MEGAN analysis of metagenomic data.. Genome Research.

[59] Aziz RK, Bartels D, Best AA, DeJongh M, Disz T (2008). The RAST Server: rapid annotations using subsystems technology.. BMC Genomics.

