# Supplementary material for: Metagenomic and Metabolic Profiling of Nonlithifying and Lithifying Stromatolitic Mats of Highborne Cay, The Bahamas
Source: PLoS One. 2012 May 25;7(5):e38229. doi: 10.1371/journal.pone.0038229 (PMC3360630; doi:10.1371/journal.pone.0038229)
Supplement: Table S1 — Carbon substrate absorbance units of stromatolitic microbial mats. Substrates were considered utilized if absorbance readings were above threshold of 50 units. Values represent mean absorbance unit for three replicate phenotypic microarrays. (DOCX) [file pone.0038229.s002.docx]

| **Table S1. Carbon substrate absorbance units^a^ of stromatolitic microbial mats.** | | | |
| --- | --- | --- | --- |
|  | | | |
| **Carbon Substrates (n=190)** | **Type 1 Mat^b^ ± SEM** | **Type 3 Mat^b^ ± SEM** | **P-value** |
| L-Arabinose | 58.3 ± 6.3 | 123.0 ±28.7 | 0.07 |
| N-Acetyl-D-Glucosamine | 31.7 ± 4.6 | 34.3 ± 2.9 | 0.33 |
| D-Saccharic Acid | 17.3 ± 9.4 | 109.0 ± 40.8 | 0.07 |
| Succinic Acid | 1.7 ± 1.67 | 38.7 ± 4.48 | 0.00 |
| D-Galactose | 57.3 ± 17.1 | 142.0 ± 12.5 | 0.01 |
| L-Aspartic Acid | 32.3 ± 13.9 | 43.3 ± 3.9 | 0.26 |
| L-Proline | 49.3 ± 16.9 | 43.0 ± 4.2 | 0.37 |
| D-Alanine | 11.3 ± 4.4 | 20.3 ± 5.6 | 0.14 |
| D-Trehalose | 27.7 ± 7.8 | 53.3 ± 7.1 | 0.04 |
| D-Mannose | 42.3 ± 3.5 | 75.3 ± 7.9 | 0.02 |
| Dulcitol | 63.0 ± 11.6 | 71.0 ± 10.1 | 0.32 |
| D-Serine | 19.7 ± 3.0 | 21.0 ± 4.0 | 0.40 |
| D-Sorbitol | 9.7 ± 4.7 | 17.0 ± 4.5 | 0.16 |
| Glycerol | 97.7 ± 95.7 | 68.3 ± 6.7 | 0.39 |
| L-Fucose | 41.0 ± 4.5 | 67.0 ± 7.2 | 0.02 |
| D-Glucuronic Acid | 17.3 ± 5.4 | 75.3 ± 16.6 | 0.03 |
| D-Gluconic Acid | 83.3 ± 41.5 | 164.3 ± 8.4 | 0.09 |
| D,L-a-Glycerol Phosphate | 3.3 ± 1.9 | 13.7 ± 3.2 | 0.03 |
| D-Xylose | 197.7 ± 43.9 | 203.0 ± 73.5 | 0.48 |
| D,L-Lactic acid | 58.7 ± 29.4 | 57.0 ± 27.6 | 0.48 |
| Formic Acid | 7.0 ± 3.5 | 29.3 ± 10.3 | 0.08 |
| D-Mannitol | 24.7 ± 5.2 | 53.0 ± 14.0 | 0.09 |
| L-Glutamic Acid | 46.3 ± 10.7 | 64.3 ± 5.6 | 0.12 |
| D-Glucose-6-Phosphate | 25.3 ± 4.4 | 31.7 ± 4.3 | 0.18 |
| D-Galactonic Acid-g-Lactone | 2.3 ± 1.2 | 17.7 ± 3.0 | 0.01 |
| D,L-Malic Acid | 143.7 ± 37.9 | 200.0 ± 18.5 | 0.14 |
| D-Ribose | 152.3 ± 8.5 | 155.3 ± 41.5 | 0.47 |
| Tween 20 | 36.0 ± 6.7 | 58.7 ± 7.5 | 0.04 |
| L-Rhamnose | 35.0 ± 8.9 | 34.0 ± 3.1 | 0.46 |
| D-Fructose | 20.7 ± 4.7 | 50.0 ± 11.4 | 0.05 |
| Acetic Acid | 59.3 ± 1.5 | 71.3 ± 3.3 | 0.03 |
| a-D-Glucose | 41.3 ± 16.6 | 75.7 ± 14.7 | 0.10 |
| Maltose | 19.3 ± 3.8 | 67.3 ± 5.2 | 0.00 |
| D-Melibiose | 22.3 ± 2.9 | 59.3 ± 10.8 | 0.03 |
| Thymidine | 56.3 ± 4.3 | 53.0 ± 3.0 | 0.28 |
| L-Asparagine | 23.0 ± 5.2 | 29.0 ± 4.5 | 0.22 |
| D-Aspartic Acid | 9.0 ± 4.6 | 24.3 ± 3.8 | 0.03 |
| D-Glucosaminic Acid | 15.0 ± 2.9 | 23.0 ± 2.5 | 0.05 |
| 1,2-Propanediol | 16.7 ± 2.2 | 31.0 ± 3.6 | 0.02 |
| Tween 40 | 26.0 ± 4.0 | 33.7 ± 8.1 | 0.23 |
| a-Ketoglutaric Acid | 13.3 ± 3.3 | 147.7 ± 33.6 | 0.03 |
| a-Ketobutyric Acid | 14.0 ± 7.0 | 39.3 ± 13.0 | 0.09 |
| a-Methyl-D-Galactoside | 12.7 ± 1.9 | 38.3 ± 6.7 | 0.03 |
| a-D-Lactose | 25.0 ± 6.7 | 36.3 ± 11.2 | 0.22 |
| Lactulose | 22.3 ± 4.0 | 39.3 ± 5.2 | 0.03 |
| Sucrose | 31.3 ± 11.4 | 45.0 ± 10.6 | 0.21 |
| Uridine | 44.0 ± 3.0 | 47.3 ± 1.2 | 0.19 |
| L-Glutamine | 9.7 ± 0.9 | 29.0 ± 4.2 | 0.02 |
| m-Tartaric Acid | 3.7 ± 3.18 | 78.0 ± 35.1 | 0.08 |
| D-Glucose-1-Phosphate | 15.7 ± 6.12 | 22.0 ± 2.1 | 0.21 |
| D-Fructose-6-Phosphate | 24.0 ± 5.51 | 114.3 ± 75.3 | 0.18 |
| Tween 80 | 22.0 ± 2.89 | 31.7 ± 3.53 | 0.05 |
| a-Hydroxyglutaric Acid-g-Lactone | 1.7 ± 0.88 | 5.0 ± 2.52 | 0.16 |
| a-Hydroxybutyric Acid | 3.7 ± 2.33 | 40.3 ± 8.0 | 0.02 |
| b-Methyl-D-Glucoside | 21.0 ± 5.03 | 37.0 ± 5.0 | 0.04 |
| Adonitol | 9.7 ± 1.76 | 23.3 ± 6.4 | 0.08 |
| Maltotriose | 14.0 ± 1.73 | 41.0 ± 5.7 | 0.02 |
| 2'-Deoxyadenosine | 24.0 ± 1.53 | 21.0 ± 1.5 | 0.12 |
| Adenosine | 41.7 ± 20.70 | 41.0 ± 13.8 | 0.49 |
| Gly-Asp | 15.3 ± 3.76 | 24.7 ± 3.2 | 0.07 |
| Citric Acid | 119.7 ± 55.4 | 209.3 ± 45.9 | 0.14 |
| m-Inositol | 21.0 ± 2.5 | 33.0 ± 4.6 | 0.05 |
| D-Threonine | 13.7 ± 0.9 | 16.7 ± 0.3 | 0.03 |
| Fumaric Acid | 69.3 ± 35.0 | 37.3 ± 19.1 | 0.24 |
| Bromosuccinic Acid | 116.3 ± 31.7 | 162.7 ± 5.2 | 0.14 |
| Propionic Acid | 15.7 ± 2.9 | 16.0 ± 5.2 | 0.48 |
| Mucic Acid | 15.7 ± 6.2 | 90.3 ± 38.7 | 0.10 |
| Glycolic Acid | 5.0 ± 3.2 | 12.3 ± 1.2 | 0.07 |
| Glyoxylic Acid | 21.0 ± 2.5 | 21.7 ± 5.4 | 0.46 |
| D-Cellobiose | 33.7 ± 3.7 | 60.7 ± 4.9 | 0.01 |
| Inosine | 48.7 ± 2.4 | 66.0 ± 15.6 | 0.19 |
| Gly-Glu | 13.7 ± 1.8 | 21.3 ± 6.2 | 0.17 |
| Tricarballylic Acid | 0.0 ± 0 | 3.3 ± 1.7 | 0.09 |
| L-Serine | 26.3 ± 10.4 | 27.3 ± 3.3 | 0.47 |
| L-Threonine | 20.3 ± 11.4 | 43.7 ± 4.6 | 0.08 |
| L-Alanine | 14.7 ± 9.3 | 54.7 ± 14.2 | 0.04 |
| Ala-Gly | 26.7 ± 13.4 | 63.3 ± 17.7 | 0.09 |
| Acetoacetic Acid | 66.7 ± 6.5 | 62.7 ± 7.3 | 0.35 |
| N-Acetyl-D-Mannosamine | 19.3 ± 0.7 | 26.3 ± 7.2 | 0.22 |
| Mono-Methylsuccinate | 16.7 ± 13.3 | 21.3 ± 11.9 | 0.40 |
| Methylpyruvate | 104.7 ± 52.3 | 167.7 ± 1.8 | 0.18 |
| D-Malic Acid | 40.0 ± 27.0 | 123.7 ± 16.8 | 0.03 |
| L-Malic Acid | 253.7 ± 13.2 | 202.7 ± 43.8 | 0.18 |
| Gly-Pro | 14.0 ± 2.0 | 29.7 ± 2.0 | 0.00 |
| p-Hydroxyphenyl Acetic Acid | 20.6 ± 2.6 | 29.0 ± 3.8 | 0.08 |
| m-Hydroxyphenyl Acetic Acid | 31.3 ± 1.9 | 29.7 ± 3.8 | 0.36 |
| Tyramine | 27.3 ± 1.3 | 35.7 ± 8.1 | 0.21 |
| D-Psicose | 20.7 ± 5.0 | 27.7 ± 1.5 | 0.14 |
| L-Lyxose | 95.7 ± 5.5 | 99.0 ± 11.0 | 0.40 |
| Glucuronamide | 35.0 ± 4.6 | 41.7 ± 1.8 | 0.14 |
| Pyruvic Acid | 198.7 ± 79.9 | 155.3 ± 65.8 | 0.35 |
| L-Galactonic Acid-g-Lactone | 8.0 ± 4.0 | 17.7 ± 4.3 | 0.09 |
| D-Galacturonic Acid | 85.7 ± 30.0 | 122.3 ± 45.0 | 0.27 |
| b-Phenylethylamine | 25.0 ± 6.0 | 29.7 ± 3.5 | 0.28 |
| 2-Aminoethanol | 34.7 ± 3.8 | 39.3 ± 6.6 | 0.29 |
| Chondroitin Sulfate C | 26.3 ± 1.9 | 32.0 ± 0.6 | 0.04 |
| a-Cyclodextrin | 43.3 ± 4.3 | 44.7 ± 4.3 | 0.42 |
| b-Cyclodextrin | 41.3 ± 5.4 | 47.3 ± 5.0 | 0.23 |
| g-Cyclodextrin | 33.7 ± 6.7 | 43.3 ± 8.1 | 0.21 |
| Dextrin | 40.0 ± 7.6 | 43.7 ± 5.9 | 0.36 |
| Gelatin | 38.0 ± 4.1 | 68.0 ± 4.9 | 0.01 |
| Glycogen | 35.3 ± 4.9 | 39.3 ± 5.5 | 0.31 |
| Inulin | 37.3 ± 7.8 | 44.3 ± 6.2 | 0.26 |
| Laminarin | 48.7 ± 5.6 | 50.0 ± 10.4 | 0.46 |
| Mannan | 51.7 ± 7.7 | 56.0 ± 6.9 | 0.35 |
| Pectin | 168.3 ± 12.0 | 159.3 ± 41.0 | 0.43 |
| N-Acetyl-D-Galactosamine | 43.3 ± 1.8 | 44.3 ± 0.3 | 0.32 |
| N-Acetyl-Neuraminic Acid | 2.7 ± 2.7 | 88.3 ± 81.9 | 0.20 |
| b-D-Allose | 50.3 ± 5.2 | 61.3 ± 9.0 | 0.18 |
| Amygdalin | 46.3 ± 3.9 | 44.3 ± 4.8 | 0.38 |
| D-Arabinose | 81.7 ± 2.9 | 99.0 ± 4.0 | 0.01 |
| D-Arabitol | 32.7 ± 2.7 | 37.7 ± 2.7 | 0.13 |
| L-Arabitol | 31.0 ± 2.9 | 37.0 ± 2.5 | 0.10 |
| Arbutin | 32.7 ± 2.2 | 39.7 ± 2.7 | 0.06 |
| 2-Deoxy-D-Ribose | 107.0 ± 2.1 | 106.7 ± 0.7 | 0.45 |
| i-Erythritol | 33.7 ± 1.9 | 35.7 ± 2.2 | 0.26 |
| D-Fucose | 49.0 ± 4.5 | 49.7 ± 5.0 | 0.46 |
| 3-0-b-D-Galactopyranosyl-D-Arabinose | 80.7 ± 8.7 | 82.7 ± 7.5 | 0.43 |
| Gentiobiose | 39.7 ± 1.9 | 47.3 ± 0.3 | 0.02 |
| L-Glucose | 34.3 ± 1.5 | 38.7 ± 3.4 | 0.17 |
| D-Lactitol | 41.3 ± 3.4 | 40.0 ± 1.0 | 0.37 |
| D-Melezitose | 46.3 ± 1.8 | 42.7 ± 2.7 | 0.17 |
| Maltitol | 35.3 ± 3.5 | 37.0 ± 2.1 | 0.35 |
| a-Methyl-D-Galactoside | 39.0 ± 2.7 | 39.7 ± 1.5 | 0.42 |
| b-Methyl-D-Galactoside | 30.0 ± 2.7 | 34.3 ± 1.8 | 0.13 |
| 3-Methylglucose | 29.0 ± 1.0 | 38.3 ± 0.3 | 0.00 |
| b-Methyl-D-Glucuronic Acid | 27.0 ± 0.1 | 29.0 ± 2.1 | 0.22 |
| a-Methyl-D-Mannoside | 29.7 ± 1.5 | 32.7 ± 1.2 | 0.09 |
| b-Methyl-D-Xyloside | 37.3 ± 1.8 | 39.7 ± 1.2 | 0.17 |
| Palatinose | 67.0 ± 4.2 | 67.3 ± 1.2 | 0.47 |
| D-Raffinose | 43.0 ± 3.1 | 44.3 ± 2.9 | 0.38 |
| Salicin | 33.7 ± 1.5 | 33.7 ± 1.5 | 0.50 |
| Sedoheptulosan | 43.0 ± 2.0 | 37.7 ± 0.7 | 0.05 |
| L-Sorbose | 45.7 ± 6.3 | 42.3 ± 5.2 | 0.35 |
| Stachyose | 36.0 ± 1.5 | 37.7 ± 3.9 | 0.36 |
| D-Tagatose | 47.7 ± 0.9 | 51.3 ± 1.3 | 0.05 |
| Turanose | 37.3 ± 2.2 | 40.0 ± 1.7 | 0.20 |
| Xylitol | 27.7 ± 1.5 | 29.3 ± 1.2 | 0.21 |
| N-Acetyl-D-glucosaminitol | 38.3 ± 1.9 | 41.7 ± 1.2 | 0.11 |
| g-Amino-N-Butyric Acid | 27.0 ± 3.6 | 34.3 ± 0.9 | 0.09 |
| d-Amino Valeric Acid | 39.0 ± 1.0 | 40.7 ± 3.4 | 0.34 |
| Butyric Acid | 75.7 ± 5.5 | 82.0 ± 2.7 | 0.19 |
| Capric Acid | 28.0 ± 1.0 | 33.3 ± 3.8 | 0.14 |
| Caproic Acid | 39.7 ± 4.9 | 45.7 ± 4.0 | 0.20 |
| Citraconic Acid | 7.7 ± 4.1 | 9.0 ± 1.7 | 0.39 |
| D,L-Citramalic Acid | 24.7 ± 4.4 | 22.0 ± 0.6 | 0.30 |
| D-Glucosamine | 164.3 ± 10.9 | 217.3 ± 35.7 | 0.14 |
| 2-Hydroxybenzoic acid | 30.7 ± 0.7 | 32.3 ± 1.3 | 0.17 |
| 4-Hydroxybenzoic Acid | 33.0 ± 2.9 | 40.7 ± 9.5 | 0.25 |
| b-Hydroxybutyric Acid | 19.0 ± 0.6 | 22.3 ± 1.5 | 0.07 |
| g-Hydroxybutyric Acid | 34.7 ± 1.3 | 39.0 ± 1.5 | 0.05 |
| 2-Oxovaleric acid | 27.0 ± 2.1 | 32.7 ± 3.0 | 0.10 |
| Itaconic Acid | 44.0 ± 8.5 | 51.0 ± 11.6 | 0.33 |
| 5-Keto-D-Gluconic Acid | 108.0 ± 2.1 | 136.7 ± 12.6 | 0.07 |
| D-Lactic Acid Methyl Ester | 40.3 ± 2.7 | 47.3 ± 3.1 | 0.09 |
| Malonic Acid | 20.0 ± 2.7 | 21.3 ± 0.7 | 0.33 |
| Melibionic Acid | 40.3 ± 1.3 | 41.3 ± 3.0 | 0.39 |
| Oxalic Acid | 38.7 ± 7.5 | 45.7 ± 4.3 | 0.24 |
| Oxalomalic Acid | 109.7 ± 2.4 | 109.3 ± 13.0 | 0.49 |
| Quinic Acid | 32.3 ± 2.3 | 45.3 ± 2.9 | 0.01 |
| D-Ribono-1,4-Lactone | 2.3 ± 1.5 | 8.0 ± 2.7 | 0.08 |
| Sebacic Acid | 26.3 ± 0.9 | 28.3 ± 0.9 | 0.09 |
| Sorbic Acid | 72.0 ± 1.2 | 71.7 ± 0.9 | 0.42 |
| Succinamic Acid | 15.0 ± 2.1 | 20.7 ± 3.0 | 0.10 |
| D-Tartaric Acid | 26.7 ± 1.5 | 32.7 ± 3.4 | 0.11 |
| L-Tartaric Acid | 50.0 ± 2.0 | 61.0 ± 4.0 | 0.05 |
| Acetamide | 39.7 ± 3.2 | 46.7 ± 2.4 | 0.08 |
| L-Alaninamide | 43.3 ± 1.5 | 46.7 ± 1.5 | 0.09 |
| N-Acetyl-L-Glutamic Acid | 29.3 ± 1.2 | 29.7 ± 2.3 | 0.45 |
| L-Arginine | 38.3 ± 2.9 | 37.7 ± 1.2 | 0.42 |
| Glycine | 36.0 ± 3.6 | 35.7 ± 2.2 | 0.47 |
| L-Histidine | 43.3 ± 9.7 | 54.0 ± 6.1 | 0.21 |
| L-Homoserine | 32.7 ± 1.3 | 32.3 ± 0.7 | 0.42 |
| Hydroxy-L-Proline | 34.3 ± 0.9 | 38.0 ± 0.6 | 0.02 |
| L-Isoleucine | 31.3 ± 2.2 | 36.7 ± 2.2 | 0.08 |
| L-Leucine | 42.0 ± 5.2 | 50.0 ± 2.7 | 0.13 |
| L-Lysine | 44.3 ± 5.7 | 50.3 ± 4.4 | 0.23 |
| L-Methionine | 41.0 ± 9.6 | 43.7 ± 12.0 | 0.44 |
| L-Ornithine | 39.7 ± 5.2 | 43.7 ± 2.4 | 0.27 |
| L-Phenylalanine | 24.7 ± 3.7 | 32.7 ± 3.8 | 0.10 |
| L-Pyroglutamic Acid | 44.7 ± 2.3 | 46.7 ± 2.9 | 0.31 |
| L-Valine | 47.3 ± 2.2 | 49.7 ± 1.9 | 0.23 |
| D,L-Carnitine | 47.0 ± 3.5 | 50.3 ± 2.9 | 0.25 |
| Sec-Butylamine | 7.7 ± 2.3 | 15.3 ± 9.4 | 0.25 |
| D,L-Octopamine | 47.3 ± 4.3 | 48.0 ± 2.5 | 0.45 |
| Putrescine | 44.3 ± 5.2 | 44.0 ± 4.9 | 0.48 |
| Dihydroxyacetone | 178.3 ± 9.1 | 194.7 ± 8.8 | 0.13 |
| 2,3-Butanediol | 47.7 ± 6.8 | 54.7 ± 3.7 | 0.22 |
| 2,3-Butanone | 62.0 ± 4.0 | 66.0 ± 4.4 | 0.27 |
| 3-Hydroxy 2-Butanone | 61.0 ± 6.5 | 63.7 ± 6.9 | 0.40 |
| ^a^substrates were considered utilized if absorbance readings were above threshold of 50 units | | | |
| ^b^values represent mean absorbance unit for three replicate phenotypic microarrays | | | |
