# Supplementary material for: Metagenomic and Metabolic Profiling of Nonlithifying and Lithifying Stromatolitic Mats of Highborne Cay, The Bahamas
Source: PLoS One. 2012 May 25;7(5):e38229. doi: 10.1371/journal.pone.0038229 (PMC3360630; doi:10.1371/journal.pone.0038229)
Supplement: Table S2 — Nitrogen substrate absorbance units of stromatolitic microbial mats. Substrates were considered utilized if absorbance readings were above threshold of 50 units. Values represent mean absorbance unit for three replicate phenotypic microarrays. (DOCX) [file pone.0038229.s003.docx]

| **Table S2. Nitrogen substrate absorbance units^a^ of stromatolitic microbial mats** | | | |
| --- | --- | --- | --- |
|  | | | |
| **Nitrogen Substrate (n = 96)** | **Type 1 Mat^b^  ± SEM** | **Type 3 Mat^b^ ± SEM** | **P-value** |
| Ammonia | 249.7 ± 40.1 | 201.0 ± 92.5 | 0.33 |
| Nitrite | 109.7 ± 15.3 | 159.0 ± 75.1 | 0.29 |
| Nitrate | 210.6 ± 69.5 | 297.0 ± 12.5 | 0.17 |
| Urea | 192.6 ± 0.7 | 322.0 ± 7.2 | 0.00 |
| Biuret | 33.0 ± 24.0 | 17.0 ± 4.2 | 0.29 |
| L-Alanine | 278.3 ± 33.7 | 320.0 ± 3.6 | 0.17 |
| L-Arginine | 241.6 ± 39.7 | 332.3 ± 3.7 | 0.07 |
| L-Asparagine | 270.7 ± 34.8 | 327.7 ± 4.6 | 0.12 |
| L-Aspartic Acid | 289.0 ± 17.1 | 288.7 ± 3.4 | 0.49 |
| L-Cysteine | 298.3 ± 52.5 | 355.3 ± 4.1 | 0.20 |
| L-Glutamic Acid | 310.0 ± 6.9 | 324.3 ± 5.2 | 0.09 |
| L-Glutamine | 290.6 ± 30.4 | 315.0 ± 6.1 | 0.25 |
| Glycine | 186.6 ± 77.2 | 268.0 ± 14.2 | 0.20 |
| L-Histidine | 284.0 ± 19.3 | 330.3 ± 2.3 | 0.07 |
| L-Isoleucine | 37.6 ± 7.3 | 55.7 ± 2.3 | 0.06 |
| L-Leucine | 75.6 ± 37.7 | 61.0 ± 2.5 | 0.37 |
| L-Lysine | 60.0 ± 7.2 | 188.0 ± 15.5 | 0.00 |
| L-Methionine | 248.6 ± 24.8 | 150.0 ± 6.7 | 0.02 |
| L-Phenylalanine | 169.6 ± 26.4 | 176.7 ± 16.0 | 0.42 |
| L-Proline | 268.6 ± 3.2 | 256.3 ± 14.7 | 0.25 |
| L-Serine | 158.6 ± 13.3 | 159.3 ± 12.2 | 0.49 |
| L-Threonine | 189.3 ± 8.2 | 184.7 ± 35.7 | 0.45 |
| L-Tryptophan | 214.0 ± 48.2 | 280.3 ± 26.2 | 0.16 |
| L-Tyrosine | 249.3 ± 32.2 | 270.7 ± 11.4 | 0.29 |
| L-Valine | 142.3 ± 37.0 | 77.3 ±5 .3 | 0.11 |
| D-Alanine | 221.3 ± 57.6 | 264.7 ± 5.2 | 0.27 |
| D-Asparagine | 17.6 ± 0.9 | 262.7 ± 0.7 | 0.00 |
| D-Aspartic Acid | 77.3 ± 62.4 | 104.0 ± 7.5 | 0.36 |
| D-Glutamic Acid | 29.0 ± 26.5 | 276.0 ± 2.6 | 0.01 |
| D-Lysine | 9.6 ± 3.07 | 11.7 ± 4.4 | 0.36 |
| D-Serine | 102.3 ± 94.8 | 51.3 ± 4.9 | 0.32 |
| D-Valine | 33.3 ± 10.7 | 3.3 ± 1.8 | 0.05 |
| L-Citrulline | 255.3 ± 45.3 | 157.0 ± 49.6 | 0.11 |
| L-Homoserine | 62.3 ± 14.0 | 19.3 ± 2.7 | 0.04 |
| L-Ornithine | 316.3 ± 41.7 | 299.3 ± 14.3 | 0.37 |
| N-Acetyl-L-Glutamic Acid | 171.0 ± 78.0 | 285.3 ± 5.2 | 0.14 |
| N-Phthaloyl-L-Glutamic Acid | 4.3 ± 3.8 | 30.7 ± 30.2 | 0.24 |
| L-Pyroglutamic Acid | 192.0 ± 35.9 | 317.7 ± 1.9 | 0.04 |
| Hydroxylamine | 13.0 ± 1.0 | 40.3 ± 33.1 | 0.25 |
| Methylamine | 81.3 ± 17.9 | 136.3 ± 9.2 | 0.04 |
| N-Amylamine | 50.7 ± 1.2 | 92.7 ± 6.6 | 0.01 |
| N-Butylamine | 37.0 ± 15.0 | 59.7 ± 10.4 | 0.15 |
| Ethylamine | 28.7 ± 15.6 | 109.3 ± 16.3 | 0.01 |
| Ethanolamine | 240.3 ± 8.0 | 194.7 ± 21.3 | 0.08 |
| Ethylenediamine | 55.7 ± 27.6 | 7.7 ± 5.8 | 0.11 |
| Putrescine | 161.3 ± 88.5 | 214.7 ± 16.3 | 0.30 |
| Agmatine | 152.0 ± 99.0 | 247.0 ± 10.4 | 0.22 |
| Histamine | 27.7 ± 13.3 | 5.0 ± 2.0 | 0.11 |
| b-Phenylethylamine | 26.0 ± 10.0 | 2.3 ± 1.5 | 0.07 |
| Tyramine | 24.0 ± 3.2 | 19.7 ± 2.4 | 0.17 |
| Acetamide | 170.0 ± 59.6 | 130.0 ± 56.5 | 0.33 |
| Formamide | 60.7 ± 3.8 | 116.0 ± 51.6 | 0.20 |
| Glucuronamide | 198.7 ± 33.0 | 147.3 ± 21.4 | 0.14 |
| D,L-Lactamide | 8.0 ± 2.0 | 305.7 ± 4.6 | 0.00 |
| D-Glucosamine | 87.0 ± 77.0 | 152.0 ± 19.7 | 0.25 |
| D-Galactosamine | 28.7 ± 18.4 | 79.0 ± 73.5 | 0.28 |
| D-Mannosamine | 203.0 ± 53.9 | 233.7 ± 32.3 | 0.33 |
| N-Acetyl-D-Glucosamine | 297.3 ± 11.8 | 285.0 ± 4.0 | 0.21 |
| N-Acetyl-D-Galactosamine | 88.3 ± 56.1 | 16.0 ± 3.1 | 0.16 |
| N-Acetyl-D-Mannosamine | 21.7 ± 4.3 | 12.3 ± 5.2 | 0.12 |
| Adenine | 160.3 ± 29.0 | 74.3 ± 2.9 | 0.05 |
| Adenosine | 282.0 ± 15.2 | 316.0 ± 1.5 | 0.08 |
| Cytidine | 293.7 ± 16.2 | 257.3 ± 8.8 | 0.07 |
| Cytosine | 101.3 ± 15.2 | 294.0 ± 14.4 | 0.00 |
| Guanine | 169.0 ± 52.8 | 150.3 ± 23.1 | 0.38 |
| Guanosine | 244.7 ± 28.9 | 332.0 ± 2.0 | 0.05 |
| Thymine | 41.3 ± 24.9 | 119.0 ± 4.7 | 0.04 |
| Thymidine | 26.0 ± 12.6 | 122.7 ± 2.0 | 0.01 |
| Uracil | 30.3 ± 3.2 | 185.0 ± 12.6 | 0.00 |
| Uridine | 75.0 ± 18.6 | 153.7 ± 6.6 | 0.02 |
| Inosine | 313.7 ± 24.7 | 341.0 ± 2.0 | 0.19 |
| Xanthine | 266.7 ± 32.6 | 314.0 ± 5.2 | 0.14 |
| Xanthosine | 161.3 ± 24.1 | 157.7 ± 8.9 | 0.45 |
| Uric Acid | 217.7 ± 54.9 | 185.7 ± 14.5 | 0.31 |
| Alloxan | 154.0 ± 24.3 | 194.3 ± 41.4 | 0.23 |
| Allantoin | 195.7 ± 43.2 | 323.7 ± 1.5 | 0.05 |
| Parabanic Acid | 228.0 ± 26.7 | 314.3 ± 0.9 | 0.04 |
| D,L-a-Amino-N-Butyric Acid | 22.3 ± 16.3 | 69.0 ± 4.2 | 0.05 |
| g-Amino-N-Butyric Acid | 121.0 ± 48.2 | 208.3 ± 4.2 | 0.11 |
| e-Amino-N-Caproic Acid | 177.7 ± 25.2 | 177.3 ± 9.8 | 0.50 |
| D,L-a-Amino-Caprylic Acid | 63.0 ± 9.2 | 64.3 ± 2.2 | 0.45 |
| d-Amino-N-Valeric Acid | 151.0 ± 74.5 | 163.0 ± 6.9 | 0.44 |
| a-Amino-N-Valeric Acid | 119.7 ± 62.7 | 67.0 ± 4.9 | 0.24 |
| Ala-Asp | 272.0 ± 15.5 | 328.3 ± 4.7 | 0.03 |
| Ala-Gln | 324.3 ± 12.9 | 340.0 ± 5.2 | 0.18 |
| Ala-Glu | 337.0 ± 17.6 | 339.0 ± 2.9 | 0.46 |
| Ala-Gly | 338.3 ± 7.1 | 343.7 ± 0.9 | 0.26 |
| Ala-His | 251.0 ± 29.0 | 340.0 ± 4.2 | 0.04 |
| Ala-Leu | 257.3 ± 38.5 | 306.7 ± 5.2 | 0.16 |
| Ala-Thr | 244.3 ± 1.9 | 296.3 ± 5.2 | 0.00 |
| Gly-Asn | 291.3 ± 33.2 | 331.3 ± 2.8 | 0.18 |
| Gly-Gln | 274.3 ± 19.7 | 325.7 ± 5.5 | 0.06 |
| Gly-Glu | 221.3 ± 38.0 | 322.3 ± 7.5 | 0.06 |
| Gly-Met | 207.3 ± 31.2 | 297.7 ± 13.1 | 0.04 |
| Met-Ala | 301.0 ± 33.2 | 315.0 ± 9.0 | 0.36 |
| ^a^substrates were considered utilized if absorbance readings were above threshold of 50 units | | | |
| ^b^values represent mean absorbance unit for three replicate phenotypic microarrays | | | |
