# Supplementary material for: Metagenomic and Metabolic Profiling of Nonlithifying and Lithifying Stromatolitic Mats of Highborne Cay, The Bahamas
Source: PLoS One. 2012 May 25;7(5):e38229. doi: 10.1371/journal.pone.0038229 (PMC3360630; doi:10.1371/journal.pone.0038229)
Supplement: Table S3 — Phosphorus substrate absorbance units of stromatolitic microbial mats. Substrates were considered utilized if absorbance readings were above threshold of 50 units. Values represent mean absorbance unit for three replicate phenotypic microarrays. (DOCX) [file pone.0038229.s004.docx]

| **Table S3. Phosphorus substrate absorbance units^a^ of stromatolitic microbial mats.** | | | |  |
| --- | --- | --- | --- | --- |
|  | | | |  |
| **Phosphorus Substrate (n = 59)** | **Type 1 Mat^b^ ± SEM** | **Type 3 Mat^b^ ± SEM** | **P-value** |  |
| Phosphate | 298.3 ± 21.5 | 330.3 ± 7.2 | 0.14 |  |
| Pyrophosphate | 271.0 ± 21.7 | 337.3 ± 0.9 | 0.05 |  |
| Trimetaphosphate | 252.7 ± 40.4 | 333.3 ± 1.3 | 0.09 |  |
| Tripolyphosphate | 274.7 ± 38.7 | 337.0 ± 2.5 | 0.12 |  |
| Triethyl Phosphate | 33.0 ± 16.1 | 79.3 ± 12.6 | 0.05 |  |
| Hypophosphite | 98.7 ± 71.2 | 55.0 ± 3.5 | 0.30 |  |
| Adenosine 2'-Monophosphate | 338.7 ± 8.3 | 329.0 ± 6.2 | 0.20 |  |
| Adenosine 3'-Monophosphate | 256.3 ± 31.3 | 334.3 ± 1.2 | 0.07 |  |
| Adenosine 5'-Monophosphate | 299.7 ± 4.6 | 332.0 ± 2.0 | 0.01 |  |
| Adenosine 2',3'-Cyclic Monophosphate | 335.3 ± 11.7 | 338.3 ± 0.3 | 0.41 |  |
| Adenosine 3',5'-Cyclic Monophosphate | 202.7 ± 77.1 | 325.3 ± 7.2 | 0.13 |  |
| Thiophosphate | 178.3 ± 72.4 | 307.3 ± 3.8 | 0.11 |  |
| Dithiophosphate | 274.0 ± 44.5 | 328.3 ± 8.8 | 0.17 |  |
| D,L-a-Glycerol Phosphate | 270.3 ± 69.9 | 323.3 ± 1.9 | 0.26 |  |
| b-Glycerol Phosphate | 221.7 ± 2.2 | 332.0 ± 1.7 | 0.00 |  |
| Carbamyl Phosphate | 174.7 ± 85.2 | 251.0 ± 3.5 | 0.23 |  |
| D-2-Phospho-Glyceric Acid | 290.0 ± 35.6 | 319.3 ± 2.4 | 0.25 |  |
| D-3-Phospho-Glyceric Acid | 201.7 ± 16.4 | 311.7 ± 2.3 | 0.01 |  |
| Guanosine 2'-Monophosphate | 281.3 ± 8.7 | 333.0 ± 3.2 | 0.01 |  |
| Guanosine 3'-Monophosphate | 291.7 ± 7.6 | 335.7 ± 9.1 | 0.01 |  |
| Guanosine 5'-Monophosphate | 256.7 ± 33.8 | 321.7 ± 4.9 | 0.10 |  |
| Guanosine 2',3'-Cyclic Monophosphate | 310.3 ± 4.7 | 322.0 ± 11.5 | 0.21 |  |
| Guanosine 3',5'-Cyclic Monophosphate | 187.0 ± 64.2 | 295.7 ± 6.4 | 0.12 |  |
| Phosphoenol Pyruvate | 199.3 ± 25.3 | 308.0 ± 2.6 | 0.02 |  |
| Phospho-Glycolic Acid | 174.0 ± 67.5 | 298.3 ± 10.1 | 0.10 |  |
| D-Glucose-1-Phosphate | 314.0 ± 5.5 | 337.3 ± 5.5 | 0.02 |  |
| D-Glucose-6-Phosphate | 264.3 ± 47.0 | 340.7 ± 0.7 | 0.12 |  |
| 2-Deoxy-D-Glucose 6-Phosphate | 221.0 ± 21.2 | 302.3 ± 0.9 | 0.03 |  |
| D-Glucosamine-6-Phosphate | 223.7 ± 8.8 | 328.3 ± 0.9 | 0.00 |  |
| 6-Phospho-Gluconic Acid | 272.7 ± 30.3 | 315.3 ± 0.9 | 0.15 |  |
| Cytidine 2'-Monophosphate | 250.3 ± 14.1 | 253.3 ± 8.7 | 0.43 |  |
| Cytidine 3'-Monophosphate | 202.0 ± 27.2 | 283.3 ± 11.7 | 0.04 |  |
| Cytidine 5'-Monophosphate | 279.3 ± 19.8 | 313.0 ± 8.5 | 0.11 |  |
| Cytidine 2',3'-Cyclic Monophosphate | 266.7 ± 10.7 | 323.0 ± 7.5 | 0.01 |  |
| Cytidine 3',5'-Cyclic Monophosphate | 216.0 ± 65.6 | 294.7 ± 3.7 | 0.18 |  |
| D-Mannose-1-Phosphate | 296.3 ± 4.7 | 316.3 ± 1.3 | 0.02 |  |
| D-Mannose-6-Phosphate | 280.3 ± 7.2 | 318.0 ± 6.4 | 0.01 |  |
| Cysteamine-S-Phosphate | 313.0 ± 20.0 | 349.3 ± 0.3 | 0.11 |  |
| Phospho-L-Arginine | 203.0 ± 15.7 | 333.3 ± 1.9 | 0.01 |  |
| O-Phospho-D-Serine | 246.7 ± 29.2 | 275.7 ± 4.1 | 0.21 |  |
| O-Phospho-L-Serine | 194.0 ± 14.2 | 328.0 ± 1.0 | 0.00 |  |
| O-Phospho-L-Threonine | 254.0 ± 89.2 | 308.0 ± 2.1 | 0.30 |  |
| Uridine 2'-Monophosphate | 183.3 ± 51.1 | 328.7 ± 0.9 | 0.05 |  |
| Uridine 3'-Monophosphate | 231.3 ± 35.3 | 324.3 ± 3.7 | 0.06 |  |
| Uridine 5'-Monophosphate | 214.7 ± 52.8 | 318.7 ± 5.2 | 0.09 |  |
| Uridine 2',3'-Cyclic Monophosphate | 207.3 ± 48.2 | 316.7 ± 2.7 | 0.07 |  |
| Uridine 3',5'-Cyclic Monophosphate | 148.3 ± 41.8 | 298.0 ± 4.2 | 0.03 |  |
| O-Phospho-D-Tyrosine | 239.7 ± 22.5 | 315.0 ± 5.0 | 0.04 |  |
| O-Phospho-L-Tyrosine | 307.7 ± 22.5 | 332.3 ± 5.5 | 0.19 |  |
| Phosphocreatine | 291.3 ± 17.8 | 318.7 ± 0.9 | 0.13 |  |
| Phosphoryl Choline | 241.7 ± 47.7 | 312.0 ± 3.6 | 0.14 |  |
| O-Phosphoryl-Ethanolamine | 208.0 ± 65.1 | 319.7 ± 2.7 | 0.11 |  |
| Phosphono Acetic Acid | 148.7 ± 76.0 | 203.3 ± 1.8 | 0.27 |  |
| 2-Aminoethyl Phosphonic Acid | 144.7 ± 79.0 | 221.3 ± 4.3 | 0.22 |  |
| Methylene Diphosphonic Acid | 40.0 ± 113.0 | 12.3 ± 1.9 | 0.08 |  |
| Thymidine 3'-Monophosphate | 232.3 ± 35.1 | 298.3 ± 2.2 | 0.10 |  |
| Thymidine 5'-Monophosphate | 203.7 ± 41.7 | 289.7 ± 8.1 | 0.09 |  |
| Inositol Hexaphosphate | 175.0 ± 53.6 | 121.7 ± 15.6 | 0.21 |  |
| Thymidine 3',5'-Cyclic Monophosphate | 133.3 ± 41.2 | 293.7 ± 6.9 | 0.03 |  |
| ^a^substrates were considered utilized if absorbance readings were above threshold of 50 units | | | | |
| ^b^values represent mean absorbance unit for three replicate phenotypic microarrays | | | | |
