# Supplementary material for: Metagenomic and Metabolic Profiling of Nonlithifying and Lithifying Stromatolitic Mats of Highborne Cay, The Bahamas
Source: PLoS One. 2012 May 25;7(5):e38229. doi: 10.1371/journal.pone.0038229 (PMC3360630; doi:10.1371/journal.pone.0038229)
Supplement: Table S4 — Sulfur substrate absorbance units of stromatolitic microbial mats. Substrates were considered utilized if absorbance readings were above threshold of 50 units. Values represent mean absorbance unit for three replicate phenotypic microarrays. (DOCX) [file pone.0038229.s005.docx]

| **Table S4. Sulfur substrate absorbance units^a^ of stromatolitic microbial mats.** | | | |
| --- | --- | --- | --- |
|  | | | |
| **Sulfur Substrates (n = 35)** | **Type 1 Mat^b^ ± SEM** | **Type 3 Mat^b^ ± SEM** | **P-value** |
| Sulfate | 58.3 ± 12.7 | 170.3 ± 4.7 | 0.00 |
| Thiosulfate | 43.3 ± 16.4 | 141.0 ± 8.0 | 0.01 |
| Tetrathionate | 76.7 ± 11.7 | 127.7 ± 18.3 | 0.05 |
| Thiophosphate | 26.0 ± 13.6 | 119.7 ± 20.8 | 0.01 |
| Dithiophosphate | 24.3 ± 18.1 | 124.7 ± 25.3 | 0.02 |
| L-Cysteine | 85.3 ± 32.7 | 104.3 ± 27.3 | 0.34 |
| D-Cysteine | 64.7 ± 5.2 | 84.0 ± 25.7 | 0.27 |
| Cys-Gly | 56.3 ± 20.2 | 80.3 ± 20.1 | 0.22 |
| L-Cysteic Acid | 26.7 ± 13.8 | 35.3 ± 25.5 | 0.35 |
| Cysteamine | 148.3 ± 8.4 | 126.7 ± 10.9 | 0.10 |
| L-Cysteine Sulfinic Acid | 85.7 ± 20.2 | 155.7 ± 20.7 | 0.04 |
| N-Acetyl-L-Cysteine | 61.7 ± 4.4 | 87.0 ± 21.9 | 0.18 |
| S-Methyl-L-Cysteine | 55.3 ± 13.9 | 109.0 ± 27.2 | 0.09 |
| Cystathionine | 63.3 ± 11.2 | 72.0 ± 14.7 | 0.33 |
| Lanthionine | 69.7 ± 9.7 | 67.0 ± 12.1 | 0.44 |
| Glutathione | 34.7 ± 12.4 | 97.3 ± 50.7 | 0.17 |
| D,L-Ethionine | 34.0 ± 17.5 | 32.3 ± 10.5 | 0.47 |
| L-Methionine | 66.3 ± 15.6 | 154.7 ± 13.7 | 0.01 |
| D-Methionine | 58.0 ± 19.9 | 175.3 ± 10.7 | 0.01 |
| Gly-Met | 47.3 ± 13.4 | 131.7 ± 21.4 | 0.02 |
| N-Acetyl-D,L-Methionine | 52.3 ± 16.6 | 137.7 ± 31.5 | 0.05 |
| L-Methionine Sulfoxide | 38.3 ± 7.0 | 126.7 ± 18.7 | 0.01 |
| L-Methionine Sulfone | 92.0 ± 17.2 | 26.0 ± 9.3 | 0.02 |
| L-Djenkolic Acid | 39.7 ± 16.2 | 85.7 ± 29.2 | 0.13 |
| Thiourea | 51.7 ± 13.2 | 95.3 ± 13.8 | 0.04 |
| 1-Thio-b-D-Glucose | 51.3 ± 26.3 | 118.0 ± 32.8 | 0.10 |
| D,L-Lipoamide | 137.7 ± 60.6 | 210.3 ± 20.7 | 0.18 |
| Taurocholic Acid | 56.0 ± 26.9 | 77.7 ± 13.0 | 0.26 |
| Taurine | 49.3 ± 2.8 | 77.7 ± 1.5 | 0.00 |
| Hypotaurine | 44.0 ± 3.8 | 71.7 ± 9.8 | 0.05 |
| p-Aminobenzene Sulfonic Acid | 47.7 ± 28.7 | 44.0 ± 11.0 | 0.46 |
| Butane Sulfonic Acid | 72.3 ± 10.7 | 57.0 ± 11.6 | 0.19 |
| 2-Hydroxyethane Sulfonic Acid | 70.7 ± 23.4 | 73.7 ± 10.5 | 0.46 |
| Methane Sulfonic Acid | 58.0 ± 6.7 | 73.0 ± 9.3 | 0.13 |
| Tetramethylene Sulfone | 94.0 ± 11.3 | 82.3 ± 10.9 | 0.25 |
| ^a^substrates were considered utilized if absorbance readings were above threshold of 50 units | | | |
| ^b^values represent mean absorbance unit for three replicate phenotypic microarrays | | | |
